# A series of perylene diimide cathode interlayer materials for green solvent processing in conventional organic photovoltaics

**DOI:** 10.3762/bjoc.19.119

**Published:** 2023-10-26

**Authors:** Kathryn M Wolfe, Shahidul Alam, Eva German, Fahad N Alduayji, Maryam Alqurashi, Frédéric Laquai, Gregory C Welch

**Affiliations:** 1 Department of Chemistry, University of Calgary, 2500 University Drive N.W., Calgary, Alberta, T2N 1N4, Canada; 2 King Abdullah University of Science and Technology (KAUST), KAUST Solar Center (KSC), Physical Sciences and Engineering Division (PSE), Material Science and Engineering Program (MSE), Thuwal 23955-6900, Saudi Arabia

**Keywords:** cathode interlayer materials, green solvent processing, organic photovoltaics

## Abstract

Herein, we report on the design, synthesis, physical and chemical properties, and organic photovoltaic (OPV) device performance of four new cathode interlayer (CIL) materials based on bay N-annulated perylene diimides. Starting from the previously reported N-annulated perylene diimide (PDIN-H), the N-position was functionalized with a benzyl and pentafluorobenzyl group to make PDIN-B and PDIN-FB, respectively. Similarly, starting from the previously reported cyanated N-annulated perylene diimide (CN-PDIN-H), the N-position was functionalized with a benzyl and pentafluorobenzyl group to make CN-PDIN-B and CN-PDIN-FB, respectively. The materials exhibit solubility in the green solvent, ethyl acetate, and thus were processed into thin films using ethyl acetate as the solvent. The optoelectronic properties were assessed for both solution and film, and the electrochemical properties were probed in solution. To validate the potential as electron transporting layers, each film was used in conventional OPVs as the CIL with processing from ethyl acetate, while using a bulk heterojunction (BHJ) comprised of PM6:Y6. High power conversion efficiencies (PCEs) of 13% were achieved compared to control devices using the standard PFN-Br CIL.

## Introduction

Organic photovoltaic (OPV) devices for energy harvesting or light recycling are of interest due to their low cost, fabrication via layer-by-layer printing, flexibility, and low carbon footprint [1–2]. Due to the processability of organic materials used in OPVs, the large-scale manufacturing of such devices at low cost with minimal environmental impact becomes viable, especially if processed from green solvents [3–5]. These devices have the opportunity to be integrated into buildings, automobiles, Internet of Things (IoT) devices, etc. This has motivated scientists to develop OPV technology over the past several decades, which resulted in power conversion efficiencies (PCEs) of OPVs reaching over 19% by using state-of-the-art organic photoactive materials in conjunction with hole and electron transporting “interlayers” that reside between the bulk heterojunction (BHJ) and the electrodes [6]. However, while progress has been made by increases in PCEs from ≈10% to ≈19% in the last decade, many of the materials used in OPVs suffer from low thermal and/or photostability, lengthy syntheses, high cost, and require harmful reagents for synthesis and processing. Therefore, it is necessary to design new materials with studious strategies to negate these issues and contribute to the movement for the commercialization of OPVs [7–9].

Cathode interlayers (CILs) have been recently recognized as a key component to realize highly efficient OPVs. Indeed, the highest efficiency to date reported at over 19% used a BHJ of PM6:BTP-eC9 with PFN-Br as the CIL [6]. CILs serve to increase device performance in various ways. First, polar functional groups (and induced dipole moments) serve to tune the work function of the cathode for a reduced energetic offset, thus reducing the Schottky barrier that is detrimental to device performance and long-term stability. Second, efficiency is increased by tuning the frontier molecular orbitals to block holes by a deep highest occupied molecular orbital (HOMO) and by promoting electron cascade with a deep lowest unoccupied molecular orbital (LUMO). Third, CILs prevent the donor material in the BHJ from coming in contact with the cathode, thus blocking holes and reducing recombination processes. Fourth, CILs can provide smooth surface morphologies for better contact with the cathode, as otherwise defects in the BHJ, such as pinholes, can occur [10–12]. Therefore, it is important to design CIL materials to have polar groups, appropriate FMO energetic levels, and functional groups known to promote ideal packing and intermolecular interactions with neighboring compounds.

**Figure 1:**
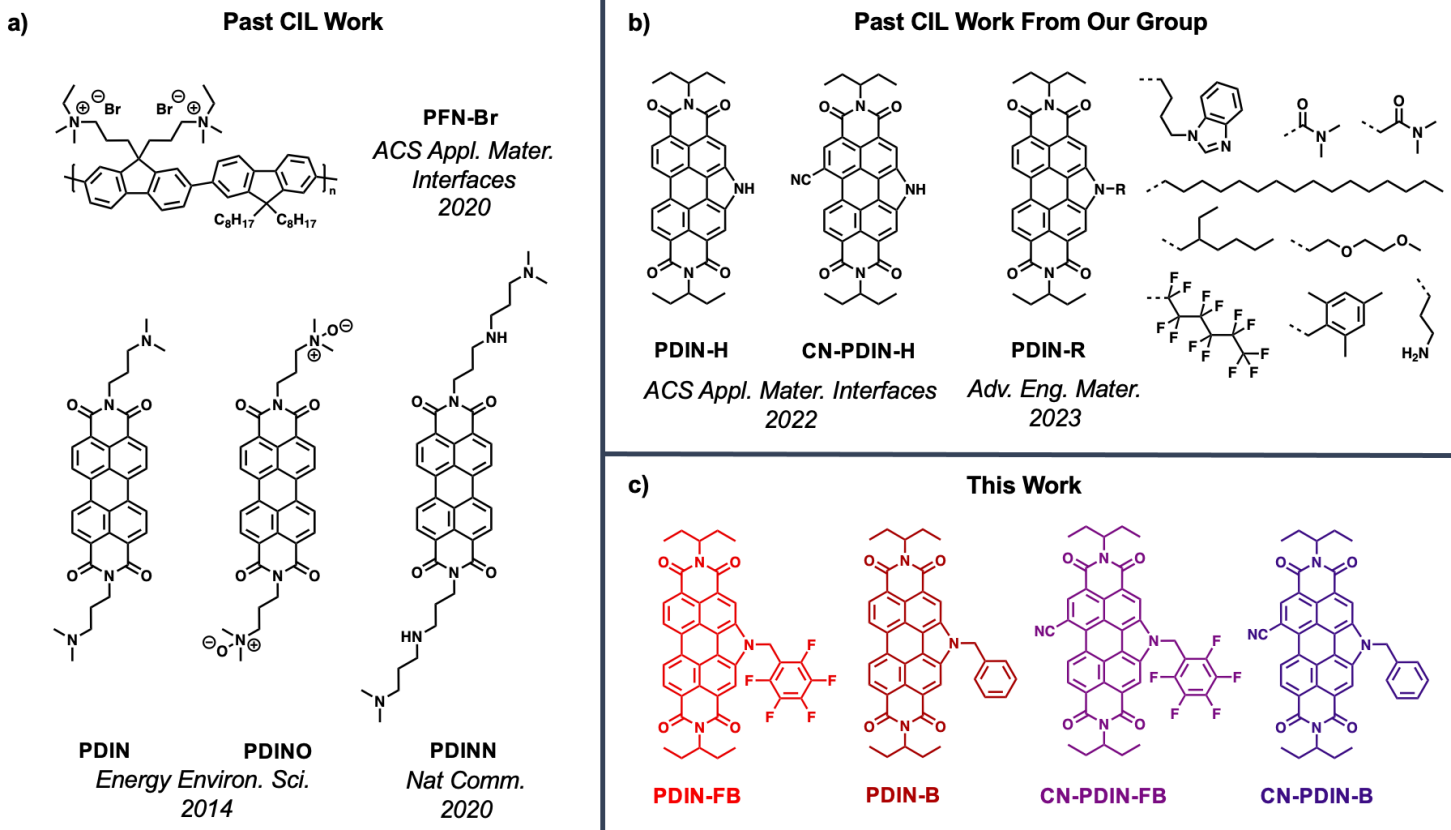
a) Select cathode interlayers used to make high-performance OPVs previously reported in the literature, including PFN-Br [13], PDIN [14], PDINO [14], and PDINN [15]. b) Cathode interlayers previously reported from our group including PDIN-H [18], CN-PDIN-H [18], and PDIN-R derivatives [19]. c) This work includes the CILs PDIN-FB, PDIN-B, CN-PDIN-FB, and CN-PDIN-B.

Several CIL compounds stand out as top-performing materials, such as PFN-Br [13], PDIN [14], PDINO [14], and PDINN [15] (Figure 1), which have realized PCEs of ≈19% [6], ≈17% [16], ≈15% [17], and ≈17% [15], respectively, when implemented as CILs in conventional OPVs. While challenges exist for polymeric materials due to batch-to-batch variations, molecular materials are advantageous as they can be easily structurally characterized, have highly reproducible syntheses, and can exhibit high solubilities in common organic solvents for effective solution processing and thin film formation. Towards obtaining high-performance CIL materials, perylene diimides (PDIs) stand out as excellent candidates as they can form electron transporting films, have appropriate LUMO energy levels compatible with most photoactive acceptor molecules (LUMO levels residing between −3.5 eV to −4.0 eV) to promote electron cascade, have appropriate HOMO energy levels at −5.5 eV and below that serve to block hole transport, exhibit high thermal stability, are highly tunable in terms of their physical and chemical properties, and can be readily doped thereby increasing electronic conductivity. Some of the most widely used PDI materials for CILs are PDIN [14], PDINN [15], and PDINO (Figure 1a) [14–15]. Past work in our group has included N-annulated PDI materials, as seen in Figure 1b, where modifications to the PDIN-H CIL material include installation of a nitrile functional group on an open bay position for electrochemical tuning, and N-functionalization to provide several different side chains to study the impact of morphological changes on device performance [18–19]. With respect to the latter, we have introduced ethyl acetate as a suitable green solvent to process CILs onto high performance BHJs (e.g., PM6:Y6). Most CILs have been processed from alcohols as to not damage the underlying hydrophobic BHJ film, but use of such alcohol-based solvents limits the types of organic materials to be used as CILs. With ethyl acetate as a processing solvent a wider range of organic materials can be developed and studied as CILs.

Herein, we report on the design, synthesis, and application of four new CILs using the previously reported N-annulated PDI (PDIN-H) and nitrile functionalized N-annulated PDI (CN-PDIN-H) compounds (Figure 1c) as the scaffolds for modification [18]. The PDIN-H scaffold was modified by N-functionalization with a benzyl (PDIN-B) or pentafluorobenzyl group (PDIN-FB). Similarly, the CN-PDIN-H scaffold was functionalized with a benzyl (CN-PDIN-B) or pentafluorobenzyl group (CN-PDIN-FB). Addition of the benzyl and pentafluorobenzyl groups was to enhance solubility in green solvents, while nitrile functionalization was done to further stabilize the LUMO of the compounds. These materials are reported for the design strategy used, their synthesis, optoelectronic properties, electrochemical properties, and processability from ethyl acetate for thin film formation. Lastly, each new CIL material was tested in conventional OPVs by processing with ethyl acetate, a green solvent [20–22], where the BHJ used was PM6:Y6 and PCEs were found to be comparable to that of PFN-Br with PCEs of over ≈13%.

## Results and Discussion

### Design strategy

**Figure 2:**
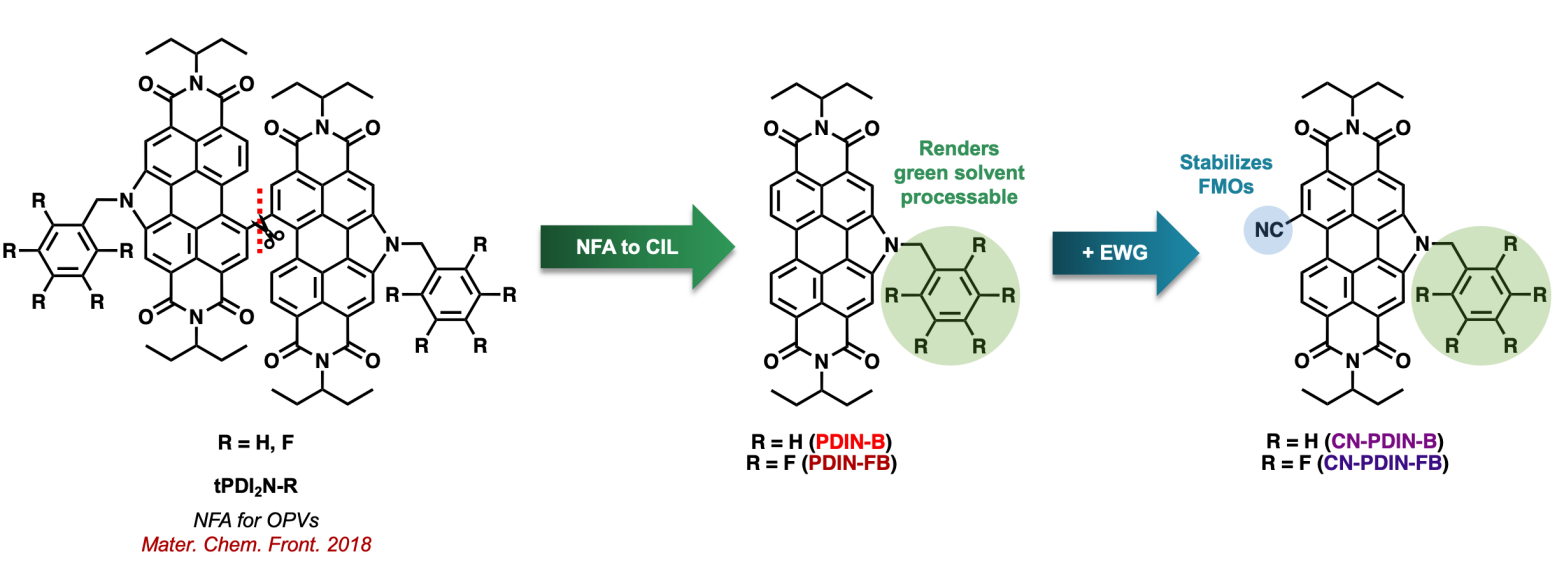
Evolution of the dimer tPDI_2_N-R [23] as an NFA to PDIN-B/PDIN-FB monomers to serve as CILs, where the addition of nitrile functional group on the monomeric PDIN-B/PDIN-FB materials serve to stabilize FMOs.

Previously, the benzyl-annulated dimers of the ethyl propyl perylene diimide (tPDI_2_-N-R; Figure 2) were reported as non-fullerene acceptors for OPVs, reaching PCEs of ≈6% [23]. Realizing that these materials are suitable electron acceptors, it was hypothesized that the monomeric species would be suitable candidates for use as CILs as the planar structure is more likely to induce ordered microstructures, which is ideal for CILs as a means to provide better contact with the cathode and BHJ acceptor materials. All derivatives (PDIN-FB, PDIN-B, CN-PDIN-FB, and CN-PDIN-B) have undergone N-functionalization to provide either a benzyl or pentafluorobenzyl group, which serves to alter the physical and chemical properties of the material. In terms of physical properties, the addition of benzyl and pentafluorobenzyl groups serve to break up NH···OH intermolecular bonding, which renders the materials soluble in a range of solvents suitable for solution processing [24]. Target organic solvents to use for the production of OPVs are those that are considered green, where a green solvent can be described as one that exhibits little to no toxicity to humans or animals, and has a minimal environmental impact when considering life-cycle assessments [21]. In terms of the electrochemical properties of the series, it was hypothesized that the addition of a nitrile functional group would stabilize both the HOMO and LUMO, where a deep-lying LUMO energy level is sought after for increased stability of electron-transporting materials [25]. Additionally, the presence of a nitrile group introduces an additional means for intermolecular bonding between the CILs and acceptor molecules (N···F and N···H bonding when using the acceptor Y6), as well as an induced dipole moment in the molecule for increased work function tuning of the cathode. The use of benzyl and pentafluorobenzyl substituents was to evaluate the impact of H vs F on the electrochemical properties and device performance.

### Synthesis

**Scheme 1:**
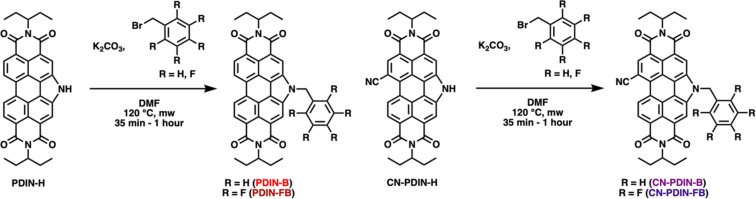
Synthesis schematic for PDIN-FB, PDIN-B, CN-PDIN-FB, and CN-PDIN-B.

The synthesis of the four CILs, PDIN-B (benzyl N-annulated ethyl propyl perylene diimide), PDIN-FB (pentafluorobenzyl N-annulated ethyl propyl perylene diimide), CN-PDIN-B (benzyl cyano N-annulated ethyl propyl perylene diimide), and CN-PDIN-FB (pentafluorobenzyl cyano N-annulated ethyl propyl perylene diimide) are reported within for the first time. Starting from PDIN-H, PDIN-B and PDIN-FB can be synthesized via N-alkylation by use of a base (K_2_CO_3_) in the presence of either benzyl bromide for PDIN-B or pentafluorobenzyl bromide for PDIN-FB (Scheme 1). Starting from CN-PDIN-H, CN-PDIN-B and CN-PDIN-FB can be synthesized by N-alkylation by use of a base (K_2_CO_3_) in the presence of either benzyl bromide for CN-PDIN-B or pentafluorobenzyl bromide for CN-PDIN-FB (Scheme 1). The products were collected by precipitating the product out of the reaction mixtures by adding a methanol/water mixture; thus, no lengthy purification steps were required for any of the syntheses. Yields of 52.4%, 80.2%, 58.1%, and 68.3% were obtained for PDIN-FB, PDIN-B, CN-PDIN-FB, and CN-PDIN-B, respectively. All compounds were structurally characterized using ^1^H NMR spectroscopy, ^13^C NMR spectroscopy, mass spectrometry, and elemental analysis. See Supporting Information File 1 for full synthetic and characterization details.

### Optical properties

**Figure 3:**
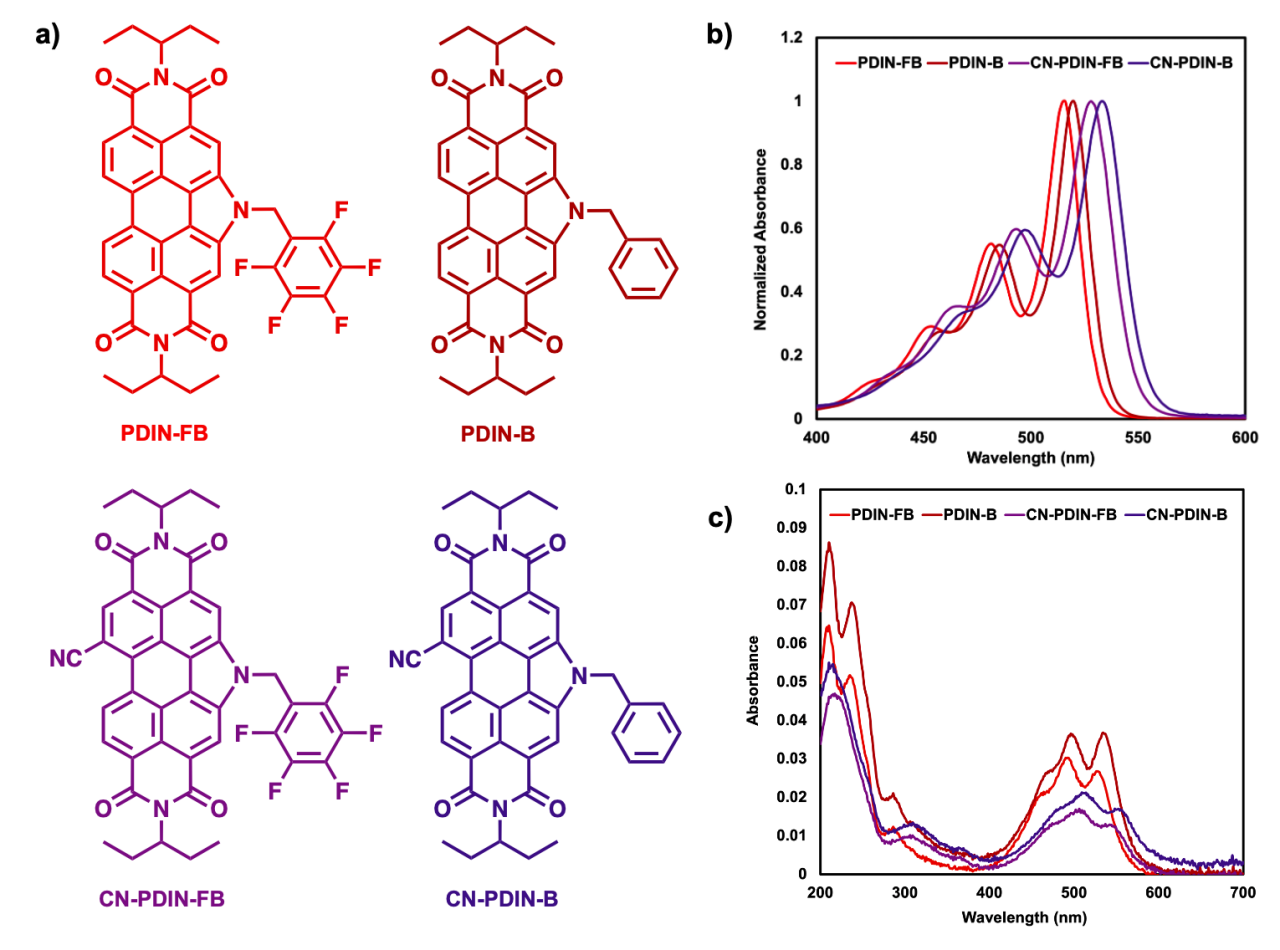
a) Structures of PDIN-FB, PDIN-B, CN-PDIN-FB, and CN-PDIN-B. b) Normalized solution UV–visible spectra using ethyl acetate as the solvent. c) UV–vis spectra for films on quartz substrates where films were cast from 1 mg/mL solutions using ethyl acetate as the solvent.

Using UV–visible spectroscopy, the optical properties for PDIN-FB, PDIN-B, CN-PDIN-FB, and CN-PDIN-FB in both solution and film form were obtained (Figure 3, Table 1), where solution spectra were done in ethyl acetate and the films were processed using ethyl acetate as the solvent and quartz as the substrate. Ethyl acetate was used due to being green and its use in OPV device fabrication (see below). When comparing the solution spectra of all the compounds, the spectrum of PDIN-FB shows an onset (in regard to the lowest energy transition at 0 → 0) at 532 nm (2.33 eV) with a λ_max_ of 515 nm, PDIN-B shows an onset of 536 nm (2.31 eV) with a λ_max_ of 520 nm, CN-PDIN-FB shows an onset of 549 nm (2.26 eV) with a λ_max_ of 528 nm, and CN-PDIN-B shows an onset of 554 nm (2.24 eV) with a λ_max_ of 533 nm. The shapes of all solution spectra are mostly retained across the compounds, which is expected due to the 0 → 0, 0 → 1, and 0 → 2 transition occurring on the perylene core [26]. In comparing the derivatives with a benzyl group on the pyrrolic position (PDIN-B, CN-PDIN-B) with those with a pentafluorobenzyl group (PDIN-FB, CN-PDIN-FB), a bathochromic shift of 0.02 eV is observed when going from the pentafluorobenzyl to the benzyl derivatives. When comparing the derivatives with (CN-PDIN-FB, CN-PDIN-B) and without (PDIN-FB, PDIN-B) a nitrile group, a bathochromic shift of 13 nm is observed for PDIN-FB → CN-PDIN-FB, and a bathochromic shift of 13 nm is observed for PDIN-B → CN-PDIN-B. The presence of the nitrile functional group results in a lowering of the energy of the optical band gap, which can be attributed to a higher degree of stabilization of the first excited state in relation to the stabilization of the ground state, which is observed in other PDIs when installing the electron-withdrawing nitrile group on the polycyclic aromatic core [27–28]. The molar extinction coefficients (ε) of all compounds in ethyl acetate were determined (see Supporting Information File 1, Figures S18–S25), where PDIN-FB has the highest ε at 85,238 M^−1^ cm^−1^, CN-PDIN-FB the second highest ε at 78,119 M^−1^ cm^−1^, PDIN-B the third highest ε at 74,489 M^−1^ cm^−1^, and CN-PDIN-B the lowest ε at 59,485 M^−1^ cm^−1^.

**Table 1:** Solution (in EtOAc) and film (on quartz) UV–visible spectra data and electrochemical data for PDIN-B, PDIN-FB, CN-PDIN-B, CN-PDIN-FB.

Compound	Solution λ_max_ (nm)	ε (M^−1^ cm^−1^)	Solution *E*_optgap_	Film *E*_optgap_	HOMO	LUMO

PDIN-FB	515	85,238	2.33 eV	2.22 eV	−5.9	−3.6
PDIN-B	520	74,489	2.31 eV	2.18 eV	−5.9	−3.6
CN-PDIN-FB	528	78,119	2.26 eV	2.13 eV	−6.1	−3.8
CN-PDIN-B	533	59,485	2.24 eV	2.09 eV	−6.1	−3.8

In terms of film spectra, all four materials were cast from a solution at a concentration of 1 mg/mL in ethyl acetate, where a concentration of 1 mg/mL was used due to CN-PDIN-FB exhibiting a maximum solubility of 1.2 mg/mL (see below). The PDIN-B film has the highest absorbance of 0.037 at its λ_max_ of 535 nm for the 0 → 0 transition and an onset of 570 nm (2.18 eV), the PDIN-FB film has the second highest absorbance of 0.030 at a λ_max_ of 495 nm for the 0 → 1 transition and an onset of 559 nm (2.22 eV), the CN-PDIN-B film has the third highest absorbance of 0.020 at a λ_max_ of 515 nm for the 0 → 1 transition and an onset of 593 nm (2.09 eV), and the CN-PDIN-FB film has the lowest absorbance of 0.017 at its λ_max_ of 509 nm for the 0 → 1 transition and an onset of 580 nm (2.13 eV). The film spectra (Figure 3) suggest that PDIN-B forms the thickest films while CN-PDIN-FB forms the thinnest films on quartz. The addition of a nitrile functional group impacts the shape of the UV–visible film spectra, leading to ill-defined 0 → 0, 0 → 1, and 0 → 2 transitions, while those without a nitrile group exhibit well-defined 0 → 0 and 0 → 1 transitions. Complex aggregation of these compounds is evident, and no clear indication of H- or J-aggregation can be concluded at this time.

### Solution processing

All four compounds are soluble in ethyl acetate, and each was probed for its saturation point. This was done by adding each material to 1 mL of ethyl acetate until solids did not dissolve, where after this each solution was filtered with a 0.22 μm PTFE filter. The absorbance of each saturated solution was measured with a UV–visible spectrophotometer to determine the concentration using the Beer–Lambert law and the previously determined molar extinction coefficients. PDIN-FB reached a saturated solution at a concentration at 8.4 mg/mL, PDIN-B a saturated solution at 19.4 mg/mL, CN-PDIN-FB a saturated solution at 3.2 mg/mL, and CN-PDIN-FB a saturated solution at 1.2 mg/mL. The compounds are also soluble in other organic solvents such as toluene, *o*-xylenes, and chloroform at concentrations of >100 mg/mL. However, in conventional OPVs, it is critical that the CIL is cast from a solvent that does not dissolve the bottom layers as keeping each layer discrete is crucial for device performance. Therefore, ethyl acetate as a processing solvent for the CILs is ideal as films of PM6:Y6 are highly solvent resistant to ethyl acetate, and because the CILs are coated onto of the BHJ in this case [19]. Furthermore, ethyl acetate is considered a green solvent due to its low toxicity and minimal associated hazards [20,22,29]. We note that each compound has minimal solubility in methanol, the most common processing solvent for CILs, and instead encourage the use of other organic materials that are soluble in ethyl acetate as CILs in conventional type OPVs. Films of each CIL on quartz substrates processed from 1 mg/mL solutions in ethyl acetate are pictured in Supporting Information File 1, Figure S26.

#### Electrochemical properties

**Figure 4:**
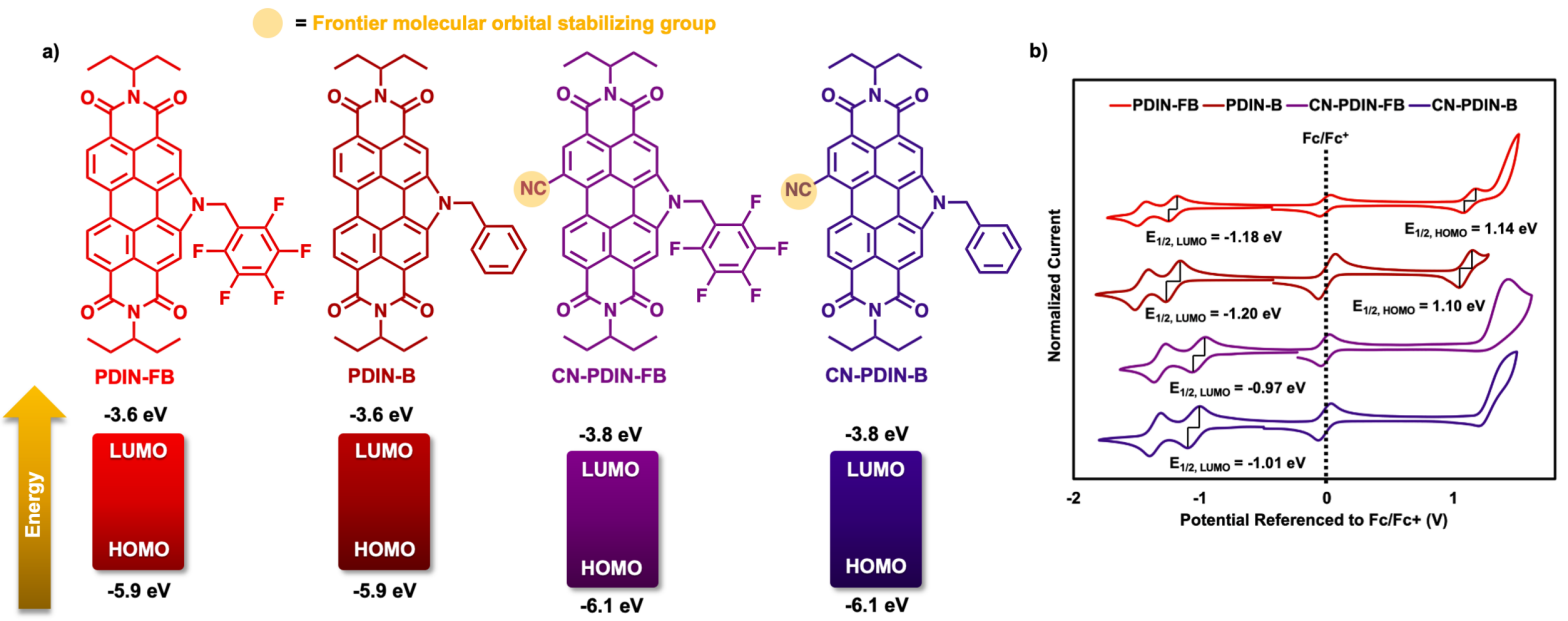
a) PDIN-FB, PDIN-B, CN-PDIN-FB, and CN-PDIN-B structures with their respective HOMO/LUMO level energies determined from CV *E*_1/2_ values. b) Corresponding CVs for PDIN-FB, PDIN-B, CN-PDIN-FB, and CN-PDIN-B with *E*_1/2_ values.

The electrochemical properties of the four CILs were probed using solution cyclic voltammetry (CV; Figure 4) and differential pulse voltammetry (DPV; Supporting Information File 1, Figures S27–S30), using dichloromethane as the solvent. For all reversible reduction or oxidation waves, HOMO and LUMO energy levels were determined using *E*_1/2_ values with Fc/Fc^+^ as the internal standard. All compounds exhibit two reversible reduction waves, where only PDIN-FB and PDIN-B exhibit a reversible oxidation wave. For CN-PDIN-FB and CN-PDIN-B, the HOMO is estimated using the optical band gap by subtracting the value in eV from the LUMO. This results in HOMO energy levels of −5.9 eV, −5.9 eV, −6.1 eV, and −6.1 eV for PDIN-FB, PDIN-B, CN-PDIN-FB, and CN-PDIN-B, respectively. Additionally, this results in LUMO energy levels of −3.6 eV, −3.6 eV, −3.8 eV, and −3.8 eV for PDIN-FB, PDIN-B, CN-PDIN-FB, and CN-PDIN-B, respectively. Both the HOMO and LUMO energy levels were confirmed using DPV, which are in agreeance with all values determined using CV (Supporting Information File 1, Figures S27–S30). The presence of a nitrile functional group stabilizes the FMOs in both CN-PDIN-FB and CN-PDIN-B by a factor of −0.2 eV (for both the HOMO and LUMO) when compared to PDIN-FB and PDIN-B, respectively. When comparing the benzyl versus pentafluorobenzyl groups, the FMOs are not significantly changed.

#### Photovoltaic device performance

**Figure 5:**
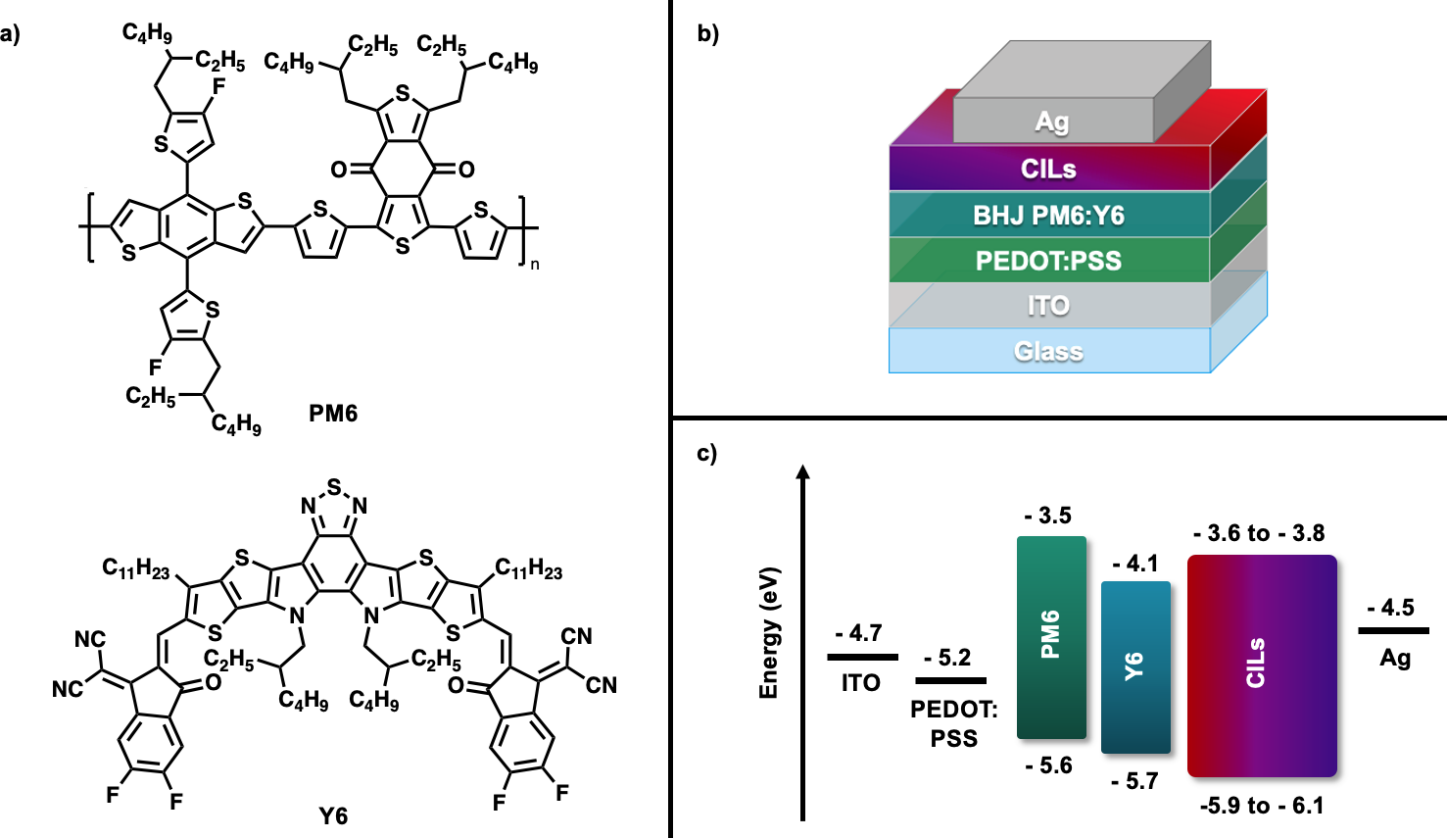
a) Chemical structures of BHJ donor material PM6 and acceptor material Y6, b) conventional OPV device structure used in this study, and c) the work functions of ITO, PEDOT:PSS, and Ag [30], as well as the energy levels of PM6, Y6, and the energy level range for the CILs used in this study.

OPV devices were fabricated using PDIN-FB, PDIN-B, CN-PDIN-FB, and CN-PDIN-B individually as CILs in conventional architecture devices with a layer stack of glass/ITO/PEDOT:PSS/PM6:Y6/CIL/Ag (Figure 5b). See Supporting Information File 1 for full device fabrication details. The BHJ materials, PM6:Y6 (Figure 5a), were selected due to their high photovoltaic performance and solvent resistance to ethyl acetate [19]. The energy level diagram of each respective layer in the device are represented in Figure 5c, where PEDOT:PSS, PM6, and Y6 work function and energy levels were taken from literature [17,30].

**Figure 6:**
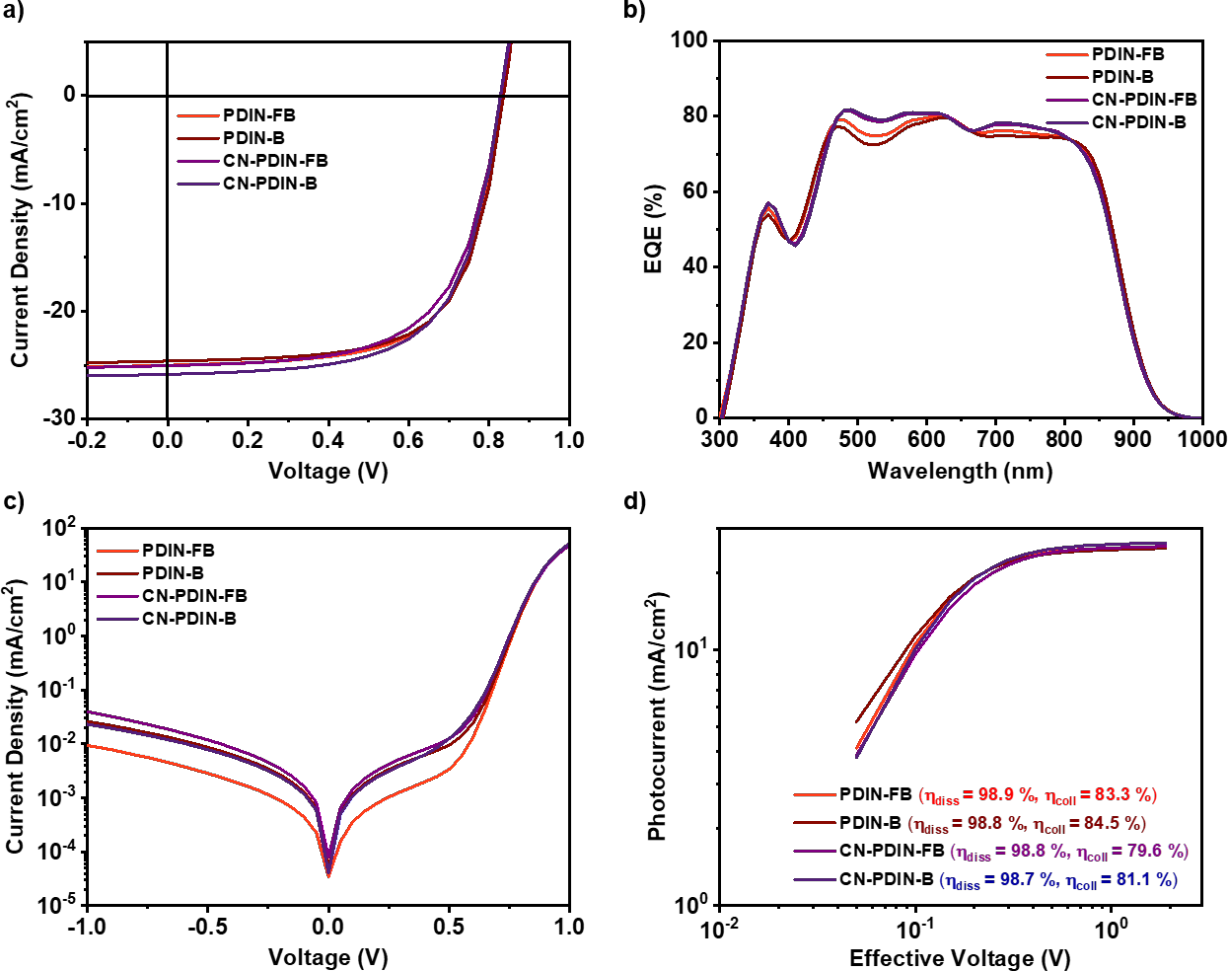
a) Current density-voltage (*I–V*) characterization under illumination, b) spectral response of the solar cells, c) *I–V* characteristics under dark, d) photocurrent vs effective voltage.

We evaluated the current density–voltage (*J–V*) in the dark and under illumination (AM1.5 G at 100 mW/cm^2^), and we also assessed the devices' external quantum efficiency (EQE), which can be seen in Figure 6a and 6b. To make the necessary adjustments to the PCE, integrated *J*_SC_ (Supporting Information File 1, Figure S31) is computed for devices with their EQE values determined [31]. The estimated values of *J*_SC_ are consistent with those measured under an illumination similar to that of the sun. Table 2 provides a summary of all of the optoelectrical and photovoltaic characteristics that were derived from the measurements of sun *I–V* and EQE. All of the devices behaved like diodes, as shown by the dark *I–V* characteristics (Figure 6c), with a rectification ratio that was higher than three orders of magnitude when comparing the current density under reverse bias and forward bias and a blocking behavior that was reasonable when the current was flowing in the opposite direction. The solar cell devices exhibit good PCEs, approximately 14%, decent FF (≈65%), and *J*_SC_ (25 mA/cm^2^). Figure S32 in Supporting Information File 1 displays the statistical evaluation of the photovoltaic (PV) parameters acquired from the *I–V* characteristics, and Supporting Information File 1, Table S1 summarizes the average PV parameters with standard deviation.

**Table 2:** Photovoltaic parameters corresponding to the OSCs varying CILs as cathode interlayers (EQE corrected). Here, the short circuit current is *J*_SC_; the open circuit voltage is *V*_OC_; the fill factor is FF, and the power conversion efficiency is η.

CIL	*J*_SC_ (mA/cm²)	*J*_SC_ (EQE) (mA/cm²)	*V*_OC_ (mV)	FF (%)	η (%)	η (EQE) (%)

PDIN-FB	24.99	**24.05**	835	65.5	13.67	**13.2**
PDIN-B	24.59	**23.85**	838	66.2	13.64	**12.4**
CN-PDIN-FB	25.02	**24.28**	831	62.9	13.07	**12.7**
CN-PDIN-B	25.807	**24.29**	831	64.1	13.74	**13.0**

All of the devices are comparable to the PFN-Br CIL, which is the one that is employed the most for PM6:Y6 OPVs. PFN-Br is most often processed from methanol [32], which is a solvent that is not well suited for the production of large-area or environmentally friendly devices owing to a high vapor pressure that can lead to precipitation during coating and due to being a highly flammable and toxic solvent [20,33–34]. Figure S33 in Supporting Information File 1 compares the *J–V* characteristics of all N-annulated perylene diimides-based CILs cast from ethyl acetate and PFN-Br cast from methanol.

Figure 6d is a plot in double logarithmic scales that depicts the photocurrent density (*J*_ph_) as a function of the effective voltage (*V*_eff_). This was conducted to investigate the charge generation and extraction processes further. The *J*_ph_ was determined by subtracting the *J–V* characteristics measured in the light from those measured in the dark using the formula *J*_ph_ = *J*_L_ – *J*_D_, where *J*_L_ denotes the current density measured in the light, and *J*_D_ represents the current density measured in the dark. The voltage (*V*_0_) that prevails when *J*_ph_ is equal to zero was used in the calculation for *V*_eff_, and the resulting value was then subtracted from the applied bias voltage (*V*_A_). The exciton dissociation efficiency (η_diss_ = *J*_SC_/*J*_sat_) and the charge collecting efficiency (η_coll_ = *J*_MPP_/*J*_sat_) were calculated based on the conditions of a short circuit and the maximum power point, respectively. Supporting Information File 1 (section 12) provides a description of each step of the calculation procedure in detail [35]. All the devices exhibited a dissociation efficiency of more than 98% and a charge collection efficiency of more than 80%, confirming the moderate *J*_SC_ and FF acquired from the device. The devices showed no barriers between the active layer and the CIL interface, indicating efficient charge extractions.

In addition, to further confirm the charge extraction and recombination, the PAIOS (platform for all-in-one characterization of solar cells) tool from Fluxim was used to measure transient photocurrent (TPC) and light intensity-dependent IV (LID-IV) measurements. TPC measurements were performed to verify charge carrier transport properties, and current decay was monitored after 500 μs pulse. The normalized TPC data for all the devices is shown in Supporting Information File 1, Figure S34a. The photocurrent decay time under short circuit conditions is very similar for all devices, and we can see that most of the charges are extracted within two μs, thus indicating more significant extraction at the interface. *J–V* measurements based on the light's intensity were carried out to determine the possible recombination process that can occur within the device. The slope (alpha) was generated from the log–log plot shown in Supporting Information File 1, Figure S34b by applying a straightforward power law dependency to the *J*_SC_ vs light intensity (I) data. This dependence was written as *J*_SC_ ∝ I^α^. The number of α near 1 suggests that non-geminate recombination does not substantially influence the *J*_SC_ at the recorded light intensity [36–37].

## Conclusion

To summarize, four new CIL materials based on N-annulated perylene diimides were synthesized, structurally characterized, probed for their physical and chemical properties, and validated as electron transporting films by implementation in conventional OPV devices. In terms of device performance, the CIL is one of the crucial parameters influencing the quality of devices' extraction and, ultimately, the FF. The scope of this article is inadequate for a detailed investigation of the processes involved in the formation of the interfacial layer between the donor–acceptor and the charge transport layer. Nevertheless, based on all the optoelectrical characterization, proposed N-annulated perylene diimides-based CILs are suitable candidates for CILs that can be replaced with any traditional transport layer and are applicable for a wide range of high-efficiency OPVs. Furthermore, the CILs processed from ethyl acetate can be applied to fabricating fully environmentally friendly OPVs based on eco-friendly or green solvents, especially for indoor applications.

## Supporting Information

File 1 Experimental part.
