# Supplementary material for: A series of perylene diimide cathode interlayer materials for green solvent processing in conventional organic photovoltaics
Source: Beilstein J Org Chem. 2023 Oct 26;19:1620–9. doi: 10.3762/bjoc.19.119 (PMC10616706; doi:10.3762/bjoc.19.119)
Supplement: File 1 — Experimental part. [file Beilstein_J_Org_Chem-19-1620-s001.pdf]

BEILSTEIN JOURNAL OF ORGANIC CHEMISTRY

Supporting Information

for

A series of perylene diimide cathode interlayer materials for green solvent processing in conventional organic photovoltaics

Kathryn M. Wolfe, Shahidul Alam, Eva German, Fahad N. Alduayji, Maryam Alqurashi, Frédéric Laquai and Gregory C. Welch

Beilstein J. Org. Chem. **2023,** *19,* 1620–1629. doi:10.3762/bjoc.19.119

Experimental part

License and Terms: This is a supporting information file under the terms of the Creative Commons Attribution License (https://creativecommons.org/licenses/by/4.0). Please note that the reuse, redistribution and reproduction in particular requires that the author(s) and source are credited and that individual graphics may be subject to special legal provisions.
The license is subject to the *Beilstein Journal of Organic Chemistry* terms and conditions: (https://www.beilstein-journals.org/bjoc/terms)

Table of Contents

1. Materials and Methods … S1
2. Synthetic Methods … S3–S5
3. ^{1}H NMR Spectra … S6–S9
4. ^{13}C NMR Spectra … S10–S13
5. Mass Spectrometry … S14–S17
6. Elemental Analysis … S18–S21
7. Molar Extinction Coefficients … S22–S25
8. Solution Processing … S26
9. Differential Pulse Voltammetry … S27–S30
10. Integrated JSC from EQE … S31
11. Statistical Evaluation of PV Parameters … S32
12. Light *J*–*V* Characteristics … S33
13. Calculation of Extraction Efficiency … S34
14. Light Intensity Dependent *J*–*V* … S34
15. References … S35

1. Materials and Methods

Materials: All reactants, reagents, and solvents were purchased from Sigma-Aldrich, Fisher Scientific, 1-material, Clevios, Organtec Ltd-BETTERCHEM, or Alfa-Aesar and were used without further purification unless otherwise indicated.

Nuclear magnetic resonance (NMR): All NMR spectroscopy experiments were recorded using a Bruker Avance 400 MHz or Bruker Avance III 600 MHz spectrometer. All experiments were performed in either $CDCl_3$ or tetrachloroethane-d_2. Chemical shifts (δ) are reported in parts per million (ppm).

High-resolution MALDI–TOF : High-resolution MALDI-TOF mass spectrometry measurements were performed by Johnson Li in the Chemical Instrumentation Facility at the University of Calgary. All spectra were acquired using a Bruker Autoflex III Smartbeam MALDI-TOF, set to the positive reflective mode (Na:YAG 355 nm laser settings: laser offset = 62–69; laser frequency = 200 Hz; and number of shots = 300). The target used was Bruker MTP 384 ground steel plate target. The sample solution (≈1 µg/mL^{-1} in dichloromethane) was mixed with matrix *trans*-2-[3-(4-*tert*-butylphenyl)-2-methyl-2-propenylidene]malononitrile (DCTB) solution (≈5 mg/mL^{-1} in methanol).

CHN elemental analysis: Elemental analysis was performed by Johnson Li in the Chemical Instrumentation Facility at the University of Calgary. A Perkin Elmer 2400 Series II CHN Elemental Analyzer was used to obtain CHN data, using ≈2.0 mg of sample.

Cyclic voltammetry (CV): The electrochemical measurements were performed using a Model 1200B Series Handheld Potentiostat by CH Instruments Inc. equipped with Ag wire, Pt wire and glassy carbon electrode, as the pseudo reference, counter electrode and working electrode respectively. Glassy carbon electrodes were polished with alumina. The cyclic voltammetry experiments were performed in anhydrous dichloromethane solution with ≈17.5 mg/mL tetrabutylammonium hexafluorophosphate ($TBAPF_6$) as the supporting electrolyte, with a ≈0.25 mg/mL concentration of the molecular material. A scan rate of 100 mV/s was used. To remove oxygen, the solutions were purged with N_2 for 15 minutes. The HOMO and LUMO energy levels were estimated by correlating the onsets of oxidation and reduction (E_{ox}Fc/Fc$^+$, E_{red}Fc/Fc$^+$) referenced to a ferrocene internal standard to that of the ionization potential of ferrocene using a conversion value of 4.8.[1]

UV–visible spectroscopy (UV–vis): All absorption measurements were recorded at room temperature using an Agilent Technologies Cary 60 optical spectrometer. All solution UV–vis spectra were measured with 1 cm quartz cuvettes, using 1-butanol as the solvent. Concentrations of 1.00×10^{-5} M, 2.00×10^{-5} M, 3.00×10^{-5} M, 4.00×10^{-5}, and 5.00×10^{-5} M were used to determine the molar extinction coefficient.

OPV device preparation: Conventional BHJ solar cells were fabricated with device configuration of ITO/PEDOT:PSS/PM6:Y6/CIL/Ag. Glass substrates with pre-patterned indium tin oxide (ITO, 16 Ω sq^{-1}) were first cleaned with the dilute detergent solution for 20 min in an ultrasonic bath. Samples were rinsed in flowing deionized water for 5 minutes, followed by a sequential bath of

acetone and isopropanol for 15 minutes each. In the next step, these cleaned samples were exposed to UV-ozone plasma cleaning for 15 min. A thin layer of PEDOT:PSS (P VP AI 4083) was used as a hole transport layer received from Clevios to facilitate charge transport. The films were annealed at 150 °C for 15 minutes to remove the residual water content from the surface and immediately transferred into the glove box. The photoactive layer solutions were prepared a day before deposition using polymer PM6 (purchased from 1-material) as donor mix with Y6 as the acceptor (purchased from Organtec Ltd-BETTERCHEM). The donor and acceptors were dissolved in chloroform (CF) at a 1:1.2 (wt.%) ratio, with an overall concentration of 15.4 mg/mL (6.16 mg of PM6 and 9.24 mg of Y6), and stirred overnight at 40 °C. As a solvent additive, 0.5 vol.% chloro-naphthalene (CN) was added to the host solution. The active layers were spin-coated over the PEDOT:PSS-coated substrates at 3000 rpm to provide a film thickness of ≈90 nm. The active layer films were annealed at 80 °C for 10 min on a pre-heated hotplate inside the glovebox. In order to improve the charge extraction, four different types of N-annulated perylene diimides-based CILs were used on top of the photoactive layer. The PDIN compounds were dissolved in ethyl acetate (EtOAc), at 0.5 mg/mL and stirred (800 rpm) overnight at room temperature. The solubility of those compounds is excellent and can be casted without any filtering. The CIL solutions were spin-cast on active layer films with 4000 rpm for 60 seconds. Afterward, the films were transferred to a thermal evaporator to evaporate 100 nm thick Ag top electrode through a shadow mask, yielding active areas of 0.1 cm^2 in each device.

Characterization of OPV devices: *J–V* measurements of solar cells were performed in the glovebox with a Keithley 2400 source meter and an Oriel Sol3A Class AAA solar simulator calibrated to one sun, AM1.5 G, with a KG-5 silicon reference cell certified by Newport. The EQE measurements were performed at zero bias by illuminating the device with a monochromatic light supplied by a Xenon arc lamp in combination with a dual-grating monochromator. Light intensity dependence *I–V* and transient photocurrent (TPC) measurements were performed using the all-in-one PAIOS 3.2 (Fluxim) measurement system. A function generator controls the light source: a white LED (rise/fall time 100 ns). A second function generator controls the applied voltage. The solar cell's current and voltage are measured with a digitizer. The current is measured via the voltage drop over a 20 Ω resistor or a trans-impedance amplifier, depending on the current amplitude.

2. Synthetic Methods

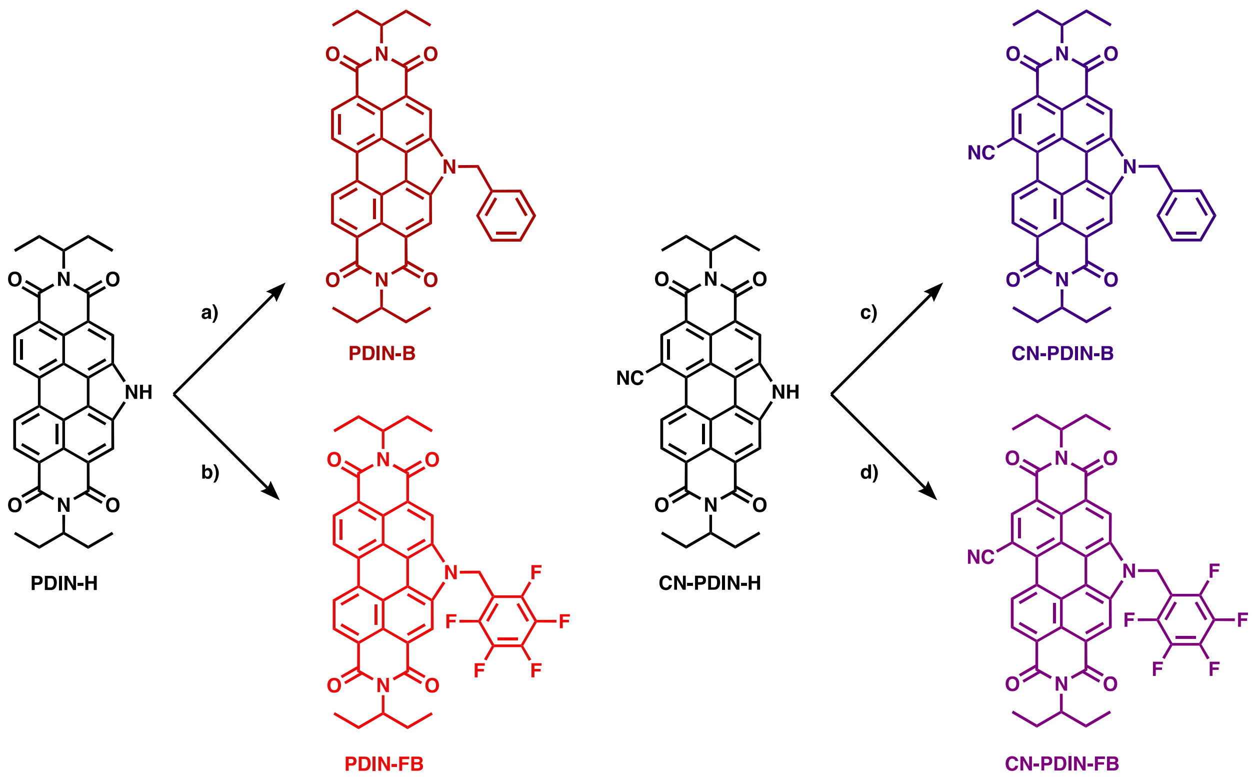

Figure S1. Synthetic reagents and conditions for PDIN-B, PDIN-FB, CN-PDIN-B, and CN-PDIN-FB. a) benzyl bromide, K_2CO_3, dimethylformamide, 120 °C, 35 minutes (80.2%); b) 2,3,4,5,6-pentafluorobenzyl bromide, K_2CO_3, dimethylformamide, 120 °C, 60 minutes in a microwave (52.4%); c) benzyl bromide, K_2CO_3, dimethylformamide, 120 °C, 35 minutes in a microwave (68.3%); d) 2,3,4,5,6-pentafluorobenzyl bromide, K_2CO_3, dimethylformamide, 120 °C, 60 minutes in a microwave (58.1%).

Synthetic procedure for PDIN-B

Into a 20 mL pressure vial, PDIN-H (100 mg; 0.184 mmol; 1 equiv) and K_2CO_3 (50.8 mg; 0.368 mmol; 2 equiv) were added. 10 mL of dimethylformamide was then added vial via cannula transfer and the reaction mixture was then purged with N_2 for 10 minutes. Benzyl bromide (0.1 mL; 0.842 mmol; 4.6 equiv) was then added via syringe and the mixture was purged with N_2 for an additional 15 minutes. The vial was then placed into a bead bath at 120 °C and was left to react for 35 minutes. Upon completion, monitored via TLC, the reaction mixture was cooled to room temperature and then poured into 200 mL of an 1:8 water:methanol solvent system and was left to stir for an hour. The solids were isolated via vacuum filtration, washing with methanol. The solids were then passed through a short silica bed using a 95% DCM with 5% methanol solvent system. The solvent was removed under vacuum, the solids slurried in methanol and then vacuum filtered to isolate a red solid (**PDIN-B**; 93.5 mg; 80.2 %).

^{1}H-NMR (400 MHz, $CDCl_3$): δ 8.96-8.93 (m, 4H), δ 8.85-8.83 (d, $^{3}J_{H-H}$ = 8 Hz, 2H), δ 7.37-7.30 (m, 5H), δ 6.05 (s, 2H), δ 5.25-5.17 (m, 2H), δ 2.40-2.29 (m, 4H), δ 2.06-1.95 (m, 4H), δ 1.00-0.96 (dd, $^{3}J_{H-H}$ = 8 Hz, 12H).
^{13}C-NMR (151 MHz, $CDCl_3$) 136.2, 135.1, 133.0, 129.3, 128.6, 127.1, 124.9, 124.1, 122.1, 120.0, 57.7, 30.9, 25.2, 11.4. *expected = 20 C; observed = 14 C.
Mass Spectrometry: ($[M-H]^+$) calculated for $C_{41}H_{35}N_3O_4$ M = 633.26; detected $[M-H]^+H^+$: 632.2575
CHN theoretical (%) C: 77.70, H: 5.57, N: 6.63; found (%) C: 76.85, H: 5.39, N: 6.33

Synthetic procedure for PDIN-FB

Into a 20 mL microwave vial, PDIN-H (100 mg; 0.184 mmol; 1 equiv) and K_2CO_3 (50.8 mg; 0.368 mmol; 2 equiv) were added. 10 mL of dimethylformamide was added to the vial via cannula transfer and the reaction mixture was then purged for 10 minutes. 2,3,4,5,6-pentafluorobenzyl bromide (0.1 mL; 0.662 mmol; 3.6 equiv) was then added via syringe and the mixture was then purged for an additional 15 minutes. The vial was then placed into a microwave set to 120 °C and was left to react for 60 minutes. Upon completion, monitored via TLC, the reaction mixture was cooled to room temperature and then poured into 200 mL of an 1:8 water:methanol solvent system and was left to stir for an hour. The solids were isolated via vacuum filtration, washing with methanol. The solids were then passed through a short silica bed using a 95% DCM with 5% methanol solvent system. The solvent was removed under vacuum, the solids slurried in methanol and then vacuum filtered to isolate a red solid (**PDIN-FB**; 69.8 mg; 52.4%).

^{1}H-NMR (400 MHz, $CDCl_3$):): δ 9.09 (s, 2H), δ 8.99-8.97 (d, $^{3}J_{H-H}$ = 8 Hz, 2H), δ 8.87-8.85 (d, $^{3}J_{H-H}$ = 8 Hz, 2H), δ 6.16 (s, 2H), δ 5.25-5.17 (m, 2H), δ 2.39-2.31 (m, 4H), δ 2.05-1.98 (m, 4H), δ 1.00-0.96 (dd, $^{3}J_{H-H}$ = 8 Hz, 12H).
^{13}C-NMR (151 MHz, $CDCl_3$) 134.8, 133.1, 125.0, 124.3, 122.0, 120.2, 57.9, 37.6, 25.2, 11.5 *expected = 20 C; observed = 10 C.
Mass Spectrometry: ($[M-H]^+$) calculated for $C_{41}H_{30}F_5N_3O_4$ M = 723.22; detected $[M-2H]^+H^+$ 722.2093
CHN theoretical (%) C: 68.05, H: 4.18, N: 5.81; found (%) C: 67.49, H: 4.00, N: 5.50

Synthetic procedure for CN-PDIN-B

Into a 20 mL pressure vial, CN-PDIN-H (100 mg; 0.176 mg; 1 equiv) and K_2CO_3 (48.5 mg; 0.352 mmol; 2 equiv) were added. 10 mL of dimethylformamide was added to the vial via cannula transfer and the reaction mixture was then purged for 10 minutes. Benzyl bromide (0.1 mL; 0.842 mmol; 4.75 equiv) was then added via syringe and the mixture was then purged for an additional 15 minutes. The vial was then placed into a bead bath at 120 °C and was left to react for 35 minutes. Upon completion, monitored via TLC, the reaction mixture was cooled to room temperature and then poured into 200 mL of a 1:8 water: methanol solvent system and was left to stir for an hour. The solids were isolated via vacuum filtration, washing with methanol. The solids were then passed through a short silica bed using a 95% DCM with 5% methanol solvent system. The solvent was removed under vacuum, the solids slurried in methanol and then vacuum filtered to isolate a red solid (**CN-PDIN-B**; 79.2 mg; 68.3%).

^{1}H-NMR (400 MHz, $CDCl_3$): δ 10.00-9.98 (d, $^3J_{H-H}$ = 8 Hz, 1H), δ 9.12-9.02 (m, 3H), δ 8.96-8.94 (d, $^3J_{H-H}$ = 8 Hz, 1H), δ 7.37-7.33 (m, 5H), δ 6.15 (s, 2H), δ 5.23-5.14 (m, 2H), δ 2.37-2.26 (m, 4H), δ 2.04-1.97 (m, 4H), δ 0.99-0.83 (m, 12H).
^{13}C-NMR (151 MHz, $CDCl_3$) 135.6, 135.2, 134.8, 133.6, 130.6, 129.3, 128.7, 127.5, 126.9, 126.3, 124.4, 121.9, 119.2, 119.0, 106.8, 58.0, 57.8, 30.7, 25.0, 24.9, 11.3, 11.2. *expected = 36 C; observed = 22 C.
Mass Spectrometry: ($[M-H]^+$) calculated for M $C_{42}H_{34}N_4O_4$ = 658.26; detected $[M-2H]^+H^+$: 657.2483
CHN theoretical (%) C: 76.58, H: 5.20, N: 8.51 found (%) C: 75.94, H: 5.08, N: 8.14

Synthetic procedure for CN-PDIN-FB

Into a 20 mL microwave vial, PDIN-H (100 mg; 0.184 mmol; 1 equiv) and K_2CO_3 (50.8 mg; 0.368 mmol; 2 equiv) were added. 10 mL of dimethylformamide was added to the vial via cannula transfer and the reaction mixture was then purged for 10 minutes. 2,3,4,5,6-pentafluorobenzyl bromide (0.1 mL; 0.662 mmol; 3.6 equiv) was then added via syringe and the mixture was then purged for an additional 15 minutes. The vial was then placed into a microwave set to 120 °C and was left to react for 60 minutes. Upon completion, monitored via TLC, the reaction mixture was cooled to room temperature and then poured into 200 mL of an 1:8 water:methanol solvent system and was left to stir for an hour. The solids were isolated via vacuum filtration, washing with methanol. The solids were then passed through a short silica bed using a 95% DCM with 5% methanol solvent system. The solvent was removed under vacuum, the solids slurried in methanol and then vacuum filtered to isolate a red solid (**CN-PDIN-FB**; 76.5 mg; 58.1%).

^{1}H-NMR (400 MHz, $CDCl_3$): δ 9.81-9.79 (d, $^3J_{H-H}$ = 8 Hz, 1H), δ 9.25 (s, 1H), δ 9.13 (s, 1H), δ 8.99 (s, 1H), δ 8.87-8.85 (d, $^3J_{H-H}$ = 8 Hz, 1H), δ 6.23 (s, 1H), δ 5.23-5.17 (m, 2H), δ 2.39-2.28 (m, 4H), δ 2.09-2.00 (m, 4H), δ 1.01-0.83 (m, 12H).
^{13}C-NMR (151 MHz, $CDCl_3$) 135.4, 134.7, 134.0, 130.9, 127.9, 126.6, 124.7, 122.1, 122.0, 119.6, 119.4, 119.2, 107.2, 58.3, 58.2, 37.7, 25.2, 25.1, 11.5, 11.4. *expected = 36 C; observed = 20 C.
Mass Spectrometry: ($[M-H]^+$) calculated for $C_{42}H_{29}F_5N_4O_4$ M = 748.21; detected $[M-2H]^+H^+$: 747.2000
CHN theoretical (%) C: 67.38, H: 3.90, N: 7.48; found (%) C: 67.43, H: 4.12, N: 7.09

3. ^{1}H NMR Spectroscopy

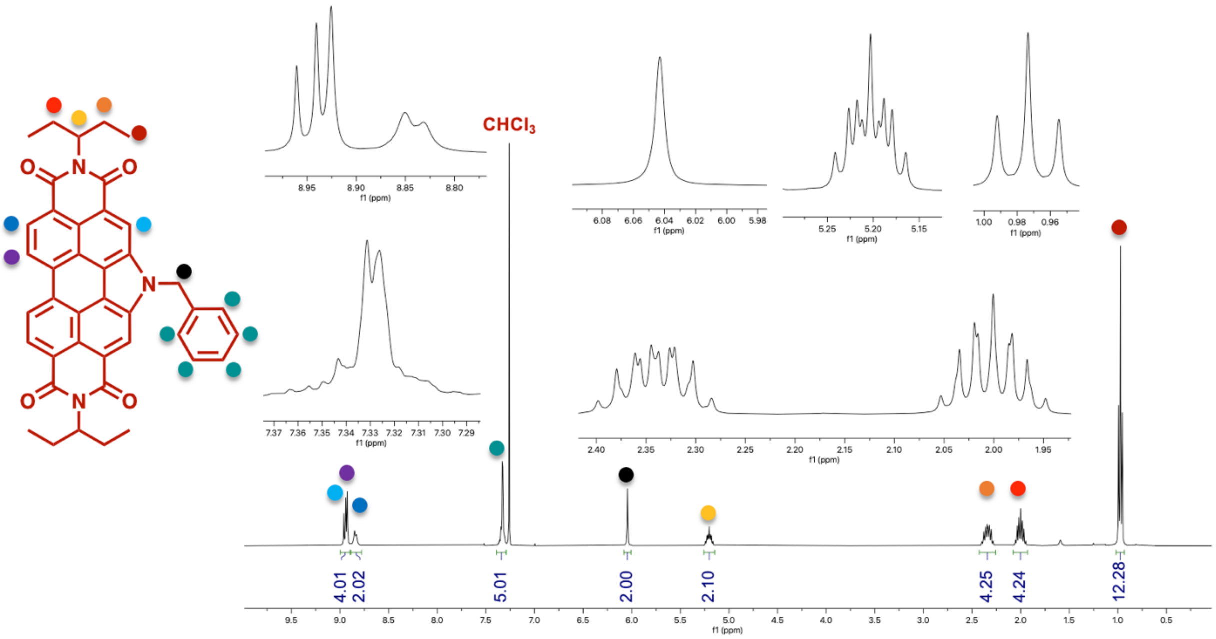

Figure S2. ^{1}H NMR spectrum of PDIN-B at 298 K in $CDCl_3$ at 400 MHz.

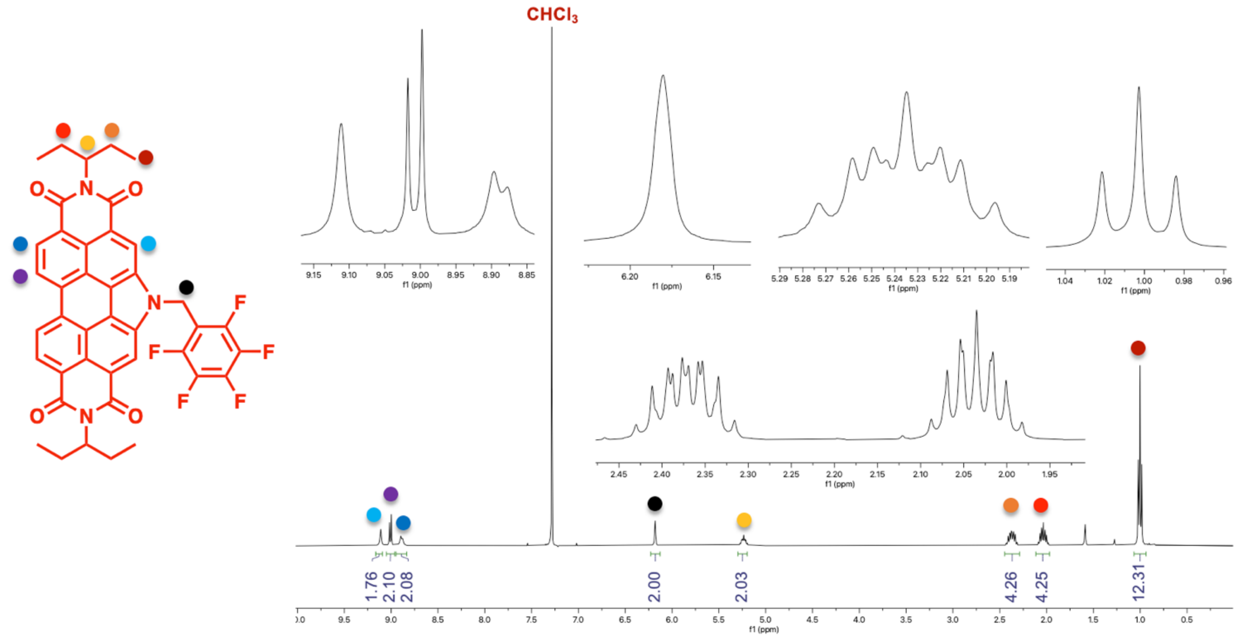

Figure S3. ^{1}H NMR spectrum of PDIN-FB at 298 K in $CDCl_3$ at 400 MHz.

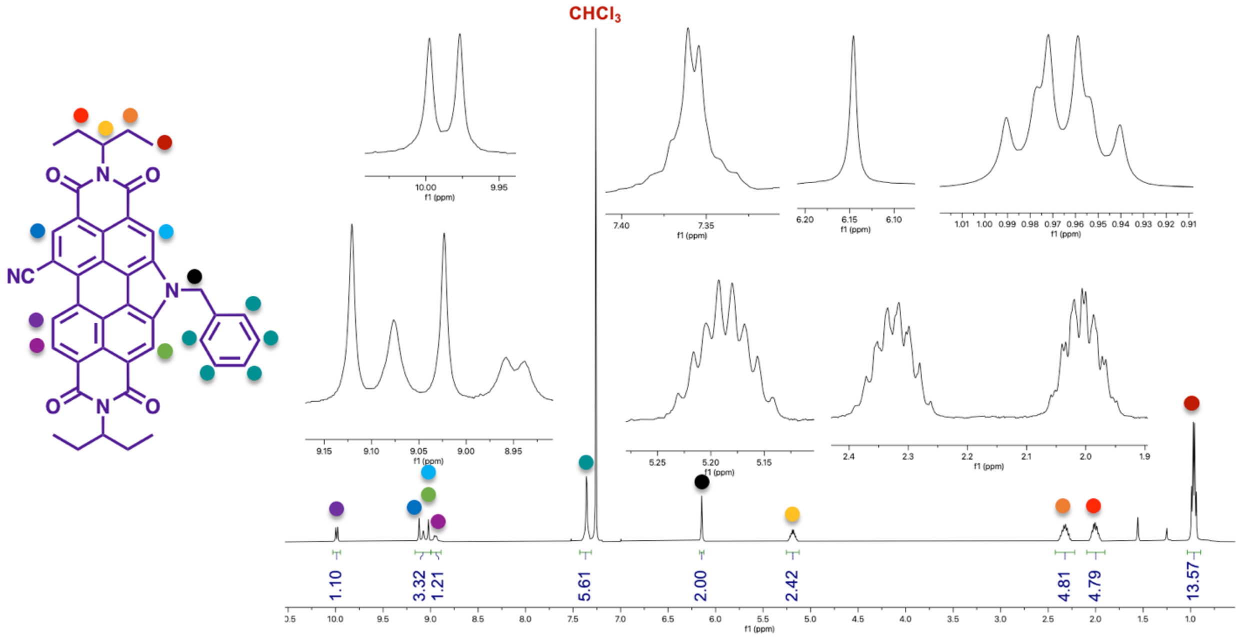

Figure S4. ^{1}H NMR spectrum of CN-PDIN-B at 298 K in $CDCl_3$ at 400 MHz.

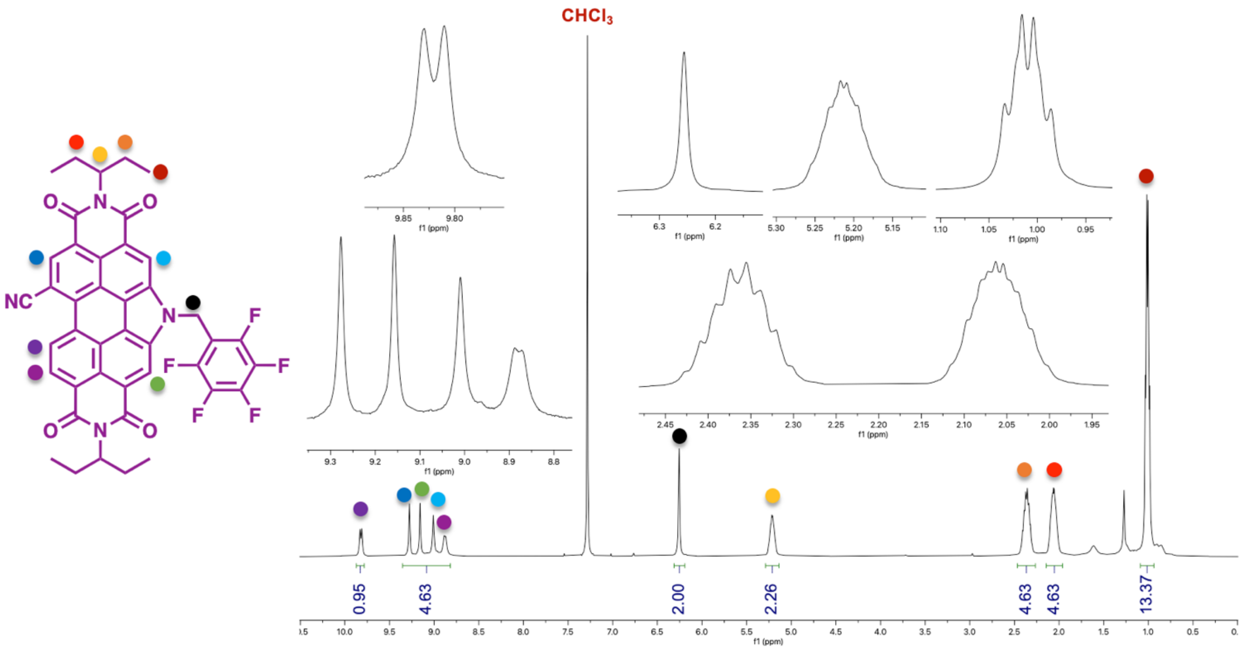

Figure S5. ^{1}H NMR spectrum of CN-PDIN-FB at 298 K in $CDCl_3$ at 400 MHz.

4. ^{13}C NMR Spectroscopy

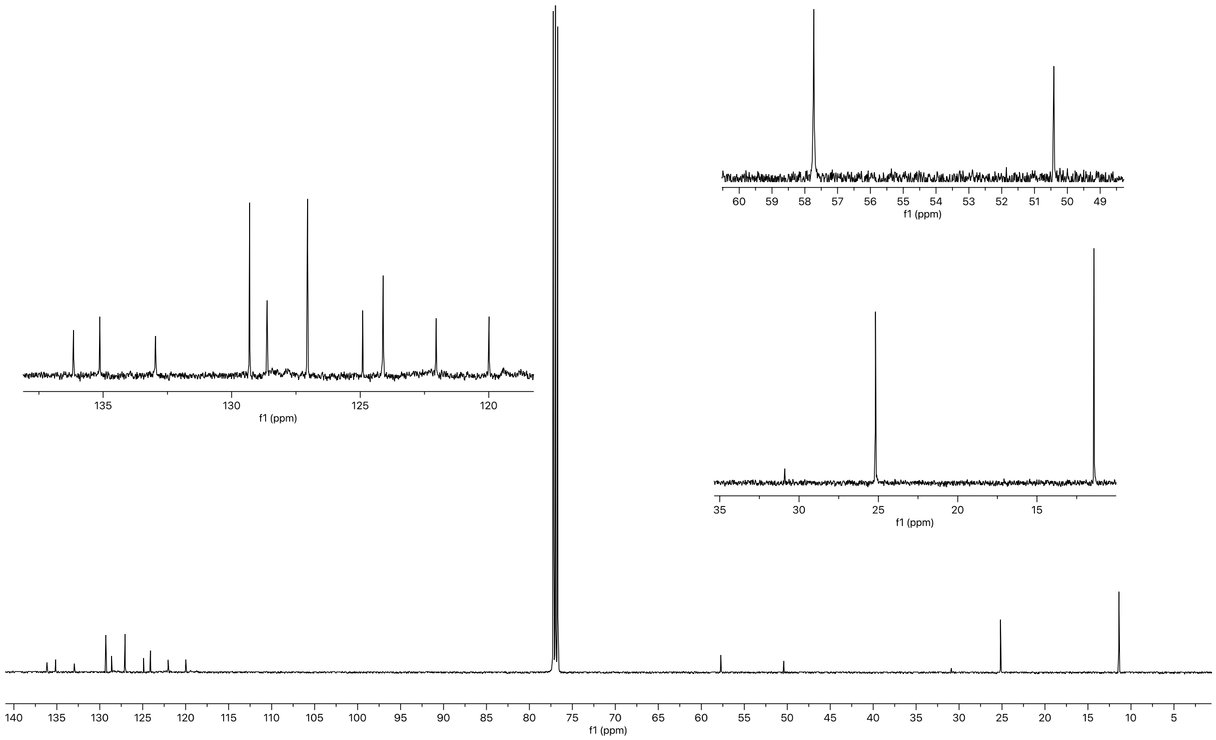

Figure S6. ^{13}C NMR spectrum of PDIN-B at 298 K in $CDCl_3$ at 151 MHz.

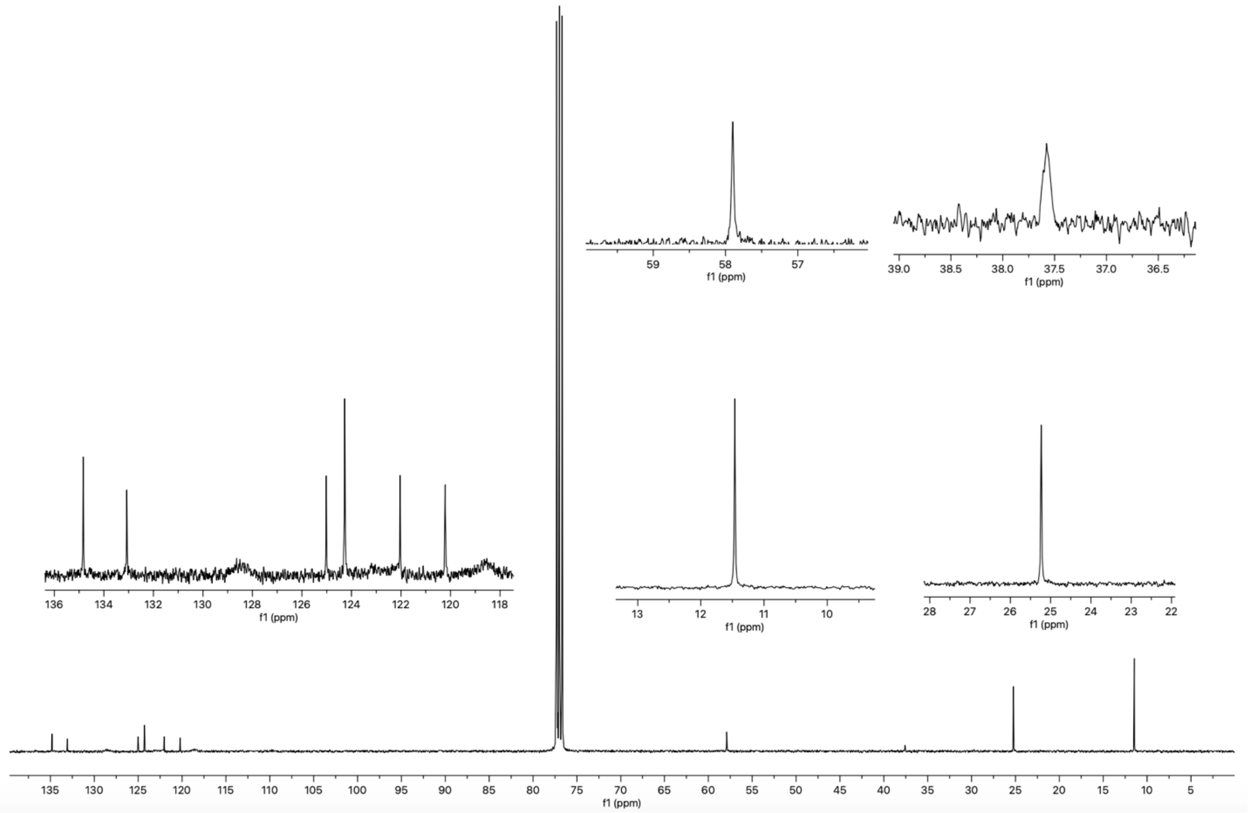

Figure S7. ^{13}C NMR spectrum of PDIN-FB at 298 K in $CDCl_3$ at 151 MHz.

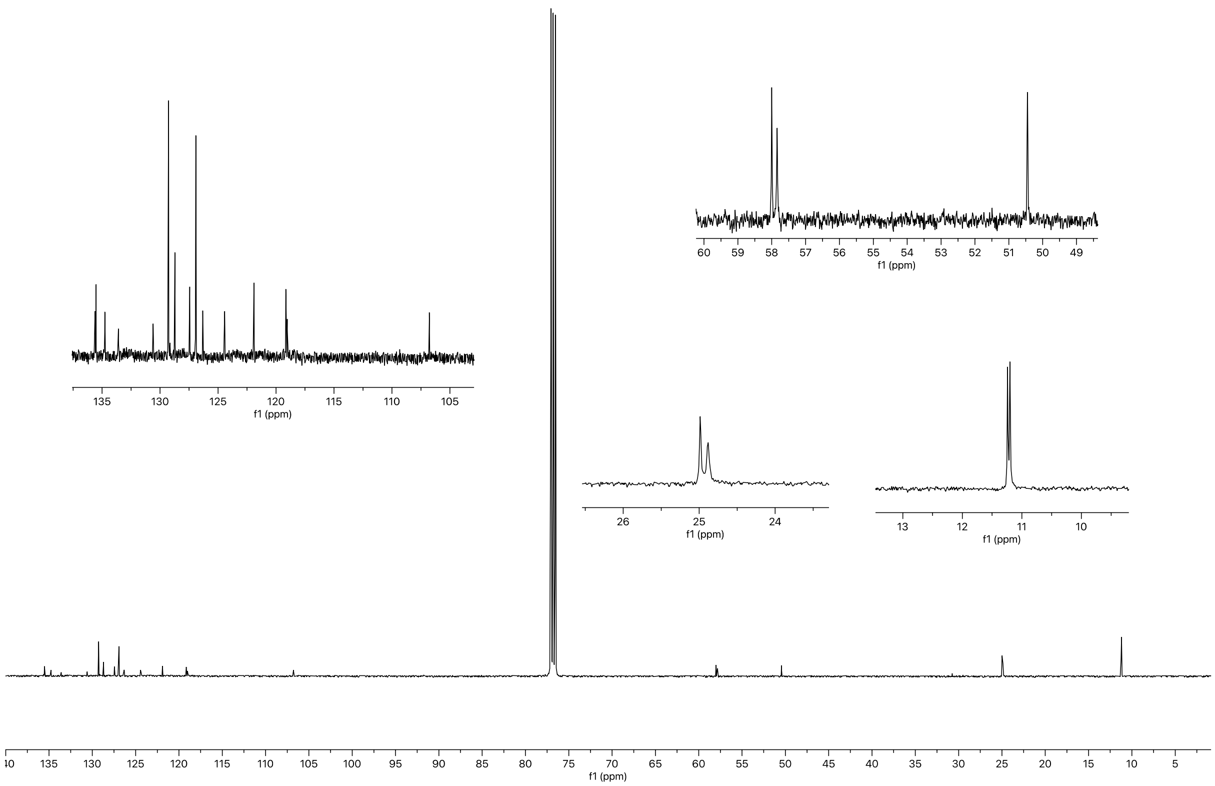

Figure S8. ^{13}C NMR spectrum of CN-PDIN-B at 298 K in $CDCl_3$ at 151 MHz.

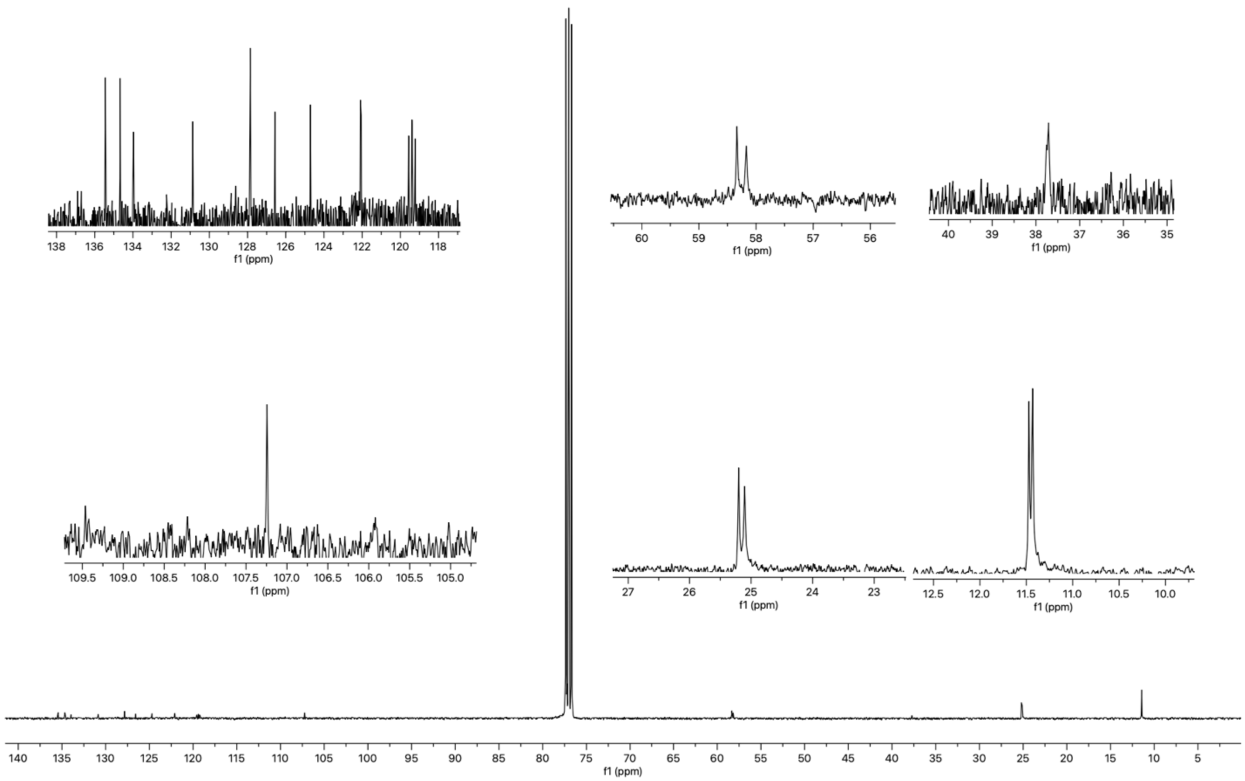

Figure S9. ^{13}C NMR spectrum of CN-PDIN-FB at 298 K in $CDCl_3$ at 151 MHz.

5. Mass Spectrometry

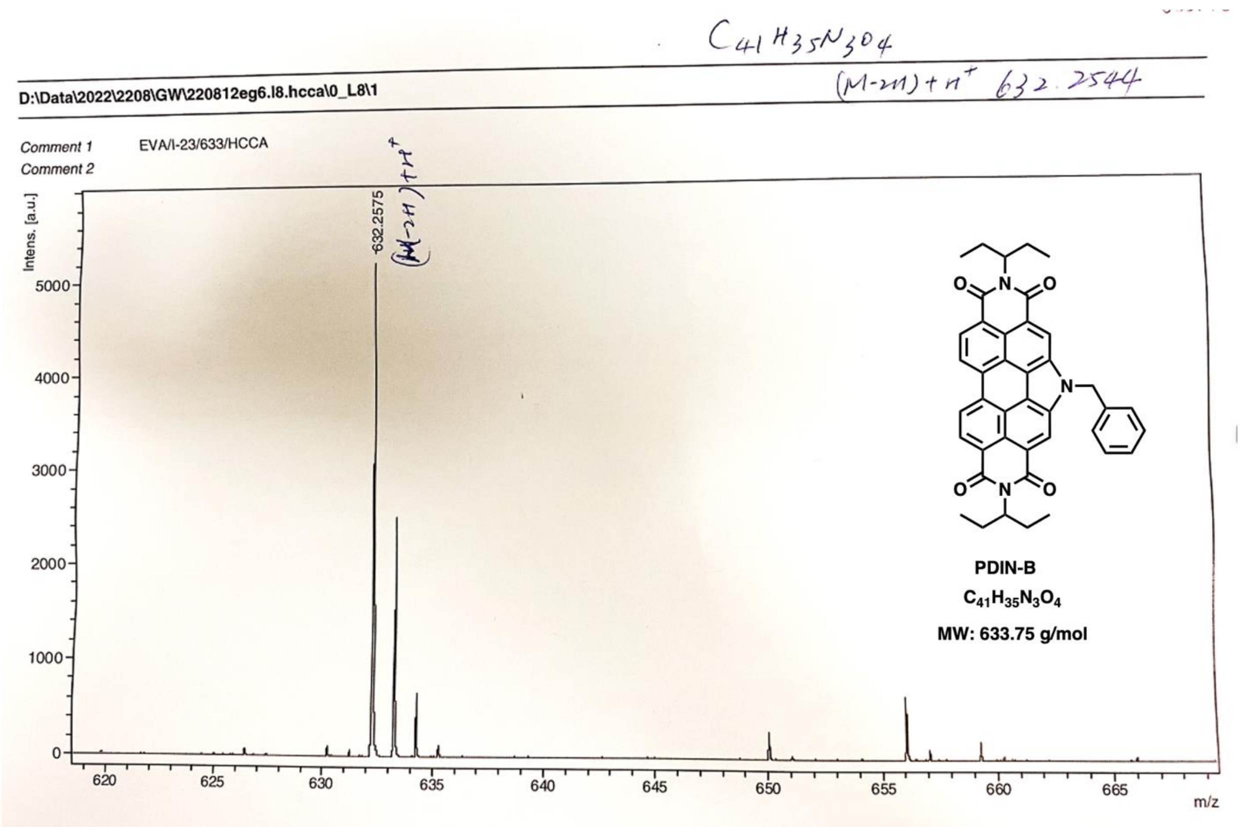

Figure S10. High-resolution MALDI-TOF spectrum for PDIN-B.

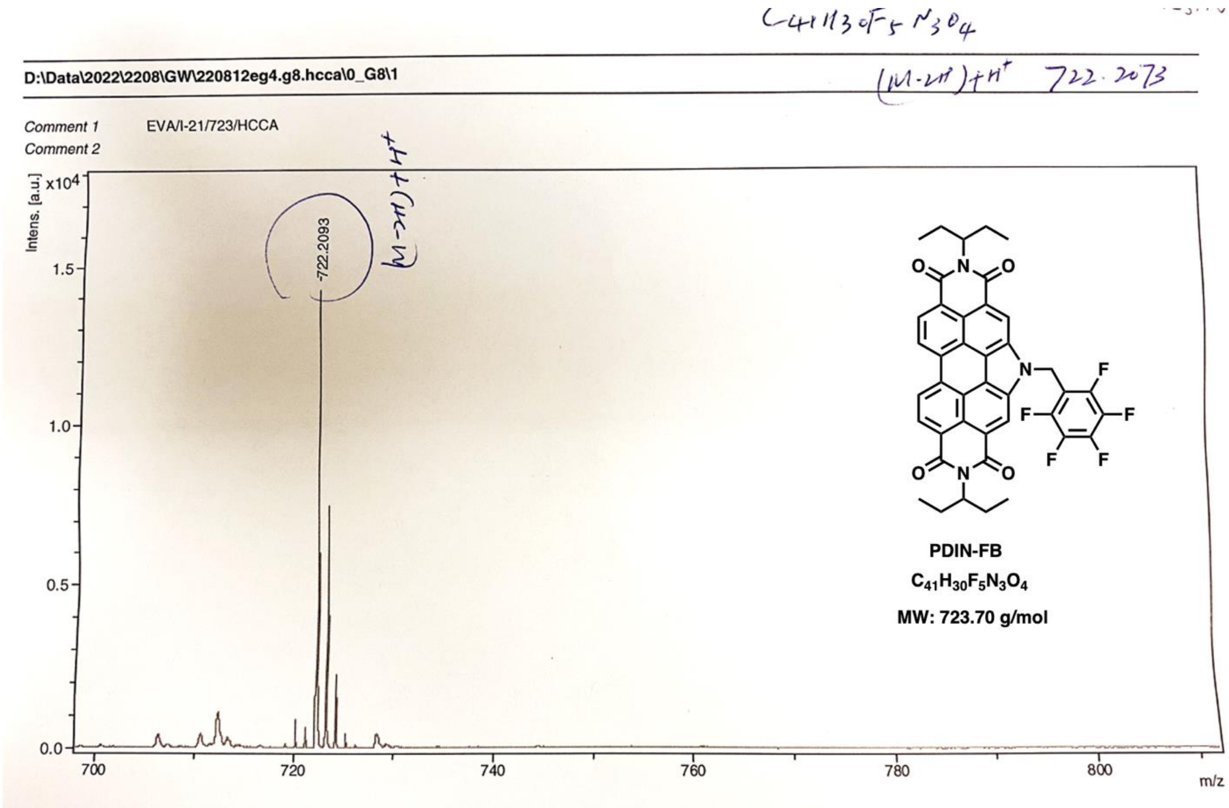

Figure S11. High-resolution MALDI-TOF spectrum for PDIN-FB.

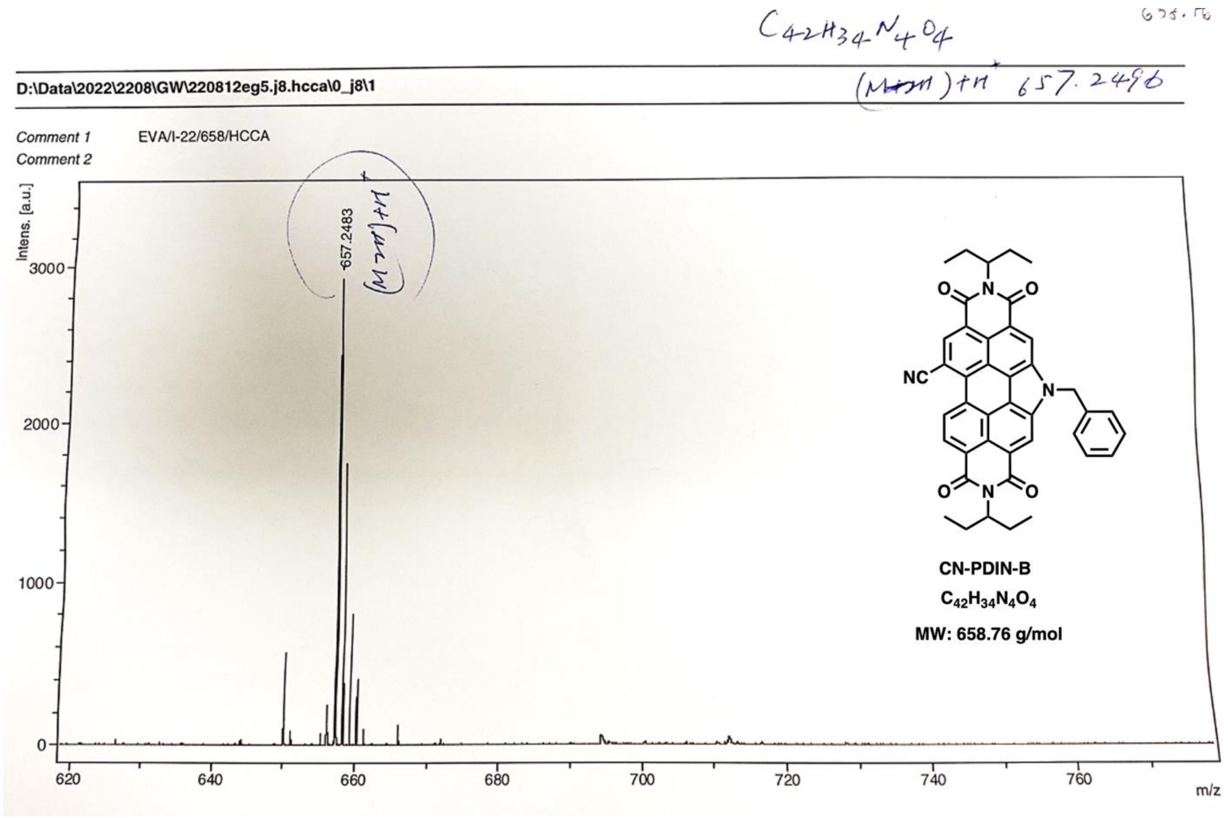

Figure S12. High-resolution MALDI-TOF spectrum for CN-PDIN-B.

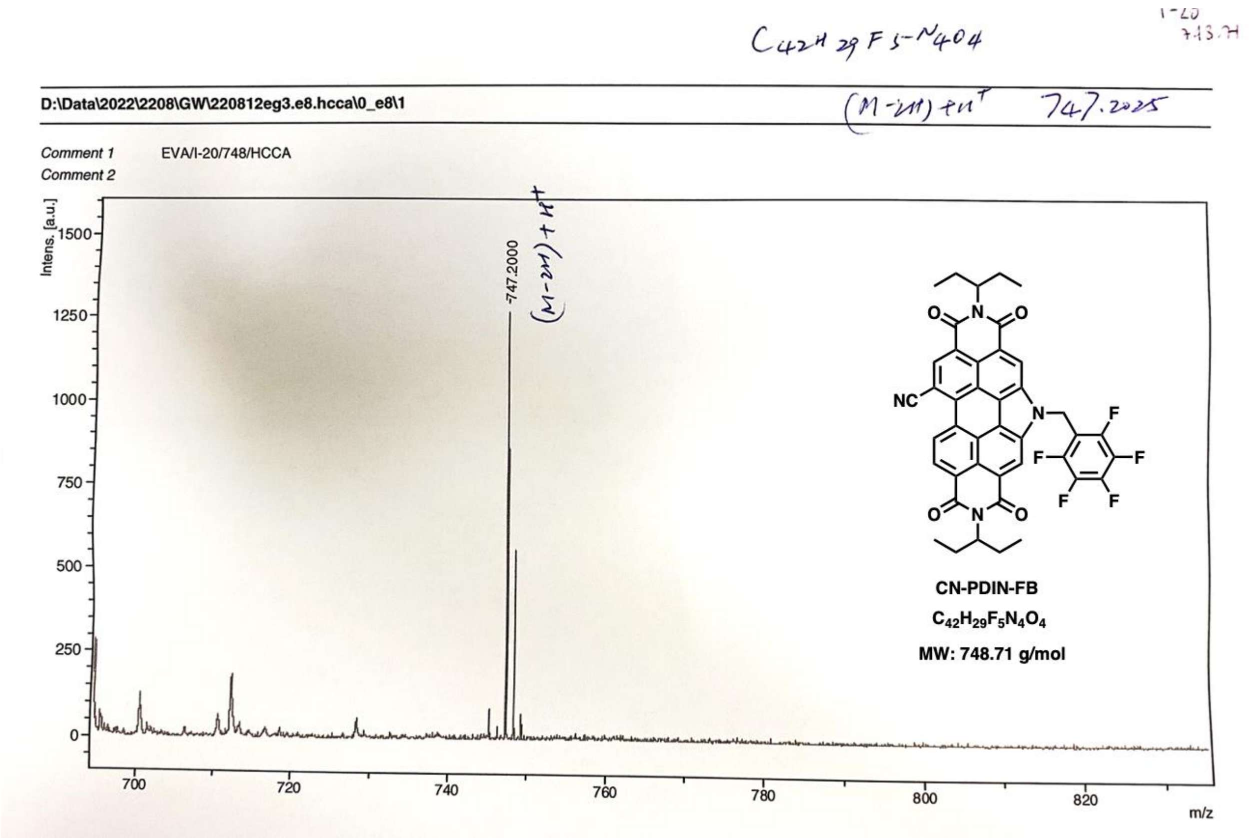

Figure S13. High-resolution MALDI-TOF spectrum for CN-PDIN-FB.

6. Elemental Analysis

University of Calgary
Department of Chemistry EA Date: 8-17-2022

Name:	EVA	Group:	GW
Sample:	EG123-2	Weight (mg):	0.964
%C (Actual):	76.85	%C (Theorectical):	77.70
%H (Actual):	5.39	%H (Theoretical):	5.57
%N (Actual):	6.33	%N (Theoretical):	6.63
%S (Actual):	0.00	%S (Theoretical):	0.00

Figure S14. Elemental analysis results for PDIN-B.

University of Calgary
Department of Chemistry EA Date: 8-17-2022

Name:	EVA	Group:	GW
Sample:	EG121-1	Weight (mg):	1.322
%C (Actual):	67.49	%C (Theorectical):	68.05
%H (Actual):	4.00	%H (Theoretical):	4.18
%N (Actual):	5.50	%N (Theoretical):	5.81
%S (Actual):	0.00	%S (Theoretical):	0.00

Figure S15. Elemental analysis results for PDIN-FB.

University of Calgary
Department of Chemistry EA Date: 8-17-2022

Name:	EVA	Group:	GW
Sample:	EG122-1	Weight (mg):	0.991
%C (Actual):	75.94	%C (Theorectical):	76.58
%H (Actual):	5.08	%H (Theoretical):	5.20
%N (Actual):	8.14	%N (Theoretical):	8.51
%S (Actual):	0.00	%S (Theoretical):	0.00

Figure S16. Elemental analysis results for CN-PDIN-B.

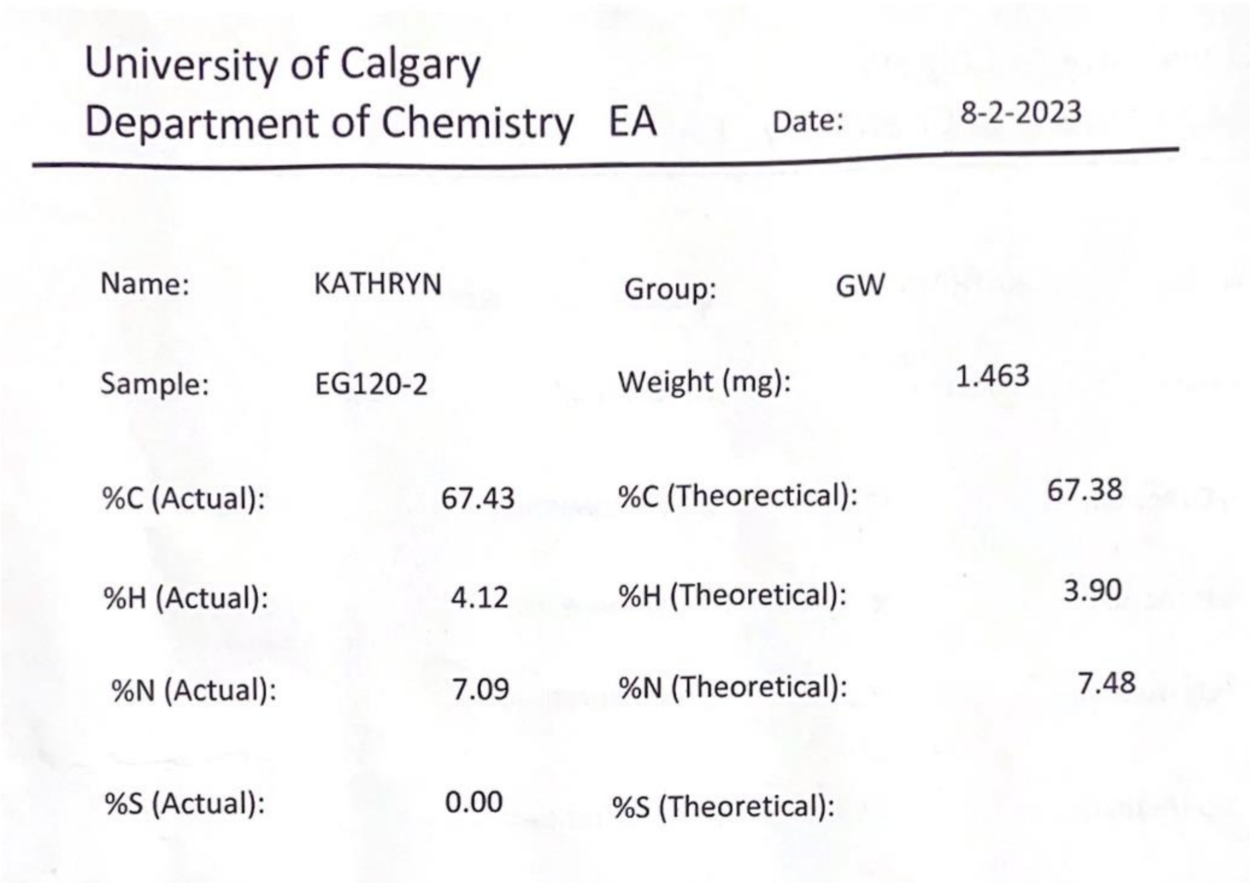

University of Calgary
Department of Chemistry EA   Date: 8-2-2023

Name:	KATHRYN	Group:	GW
Sample:	EG120-2	Weight (mg):	1.463
%C (Actual):	67.43	%C (Theorectical):	67.38
%H (Actual):	4.12	%H (Theoretical):	3.90
%N (Actual):	7.09	%N (Theoretical):	7.48
%S (Actual):	0.00	%S (Theoretical):	

Figure S17. Elemental analysis results for CN-PDIN-FB.

7. Molar Extinction Coefficients

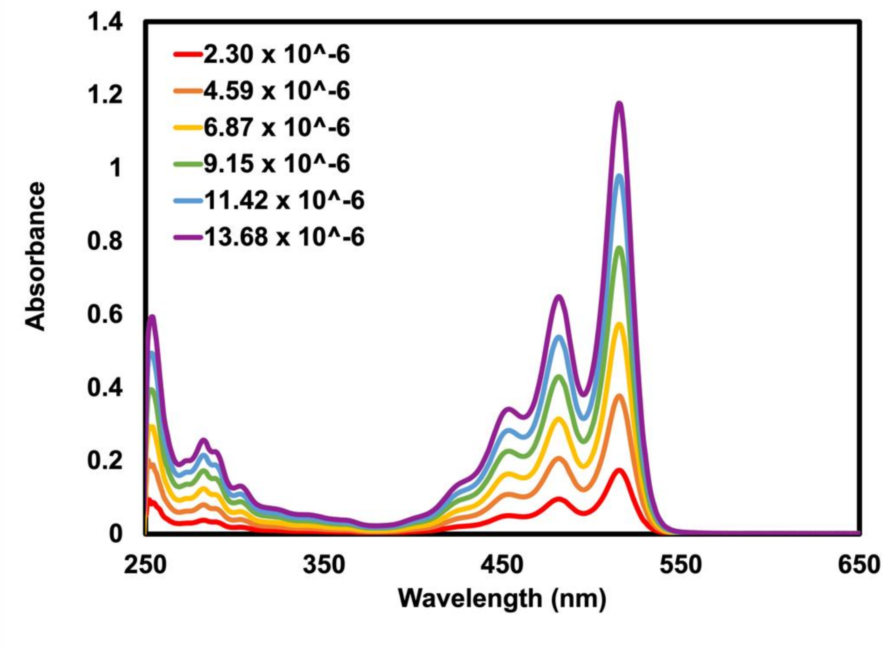

Figure S18. UV–visible spectra of PDIN-FB in ethyl acetate at different concentrations.

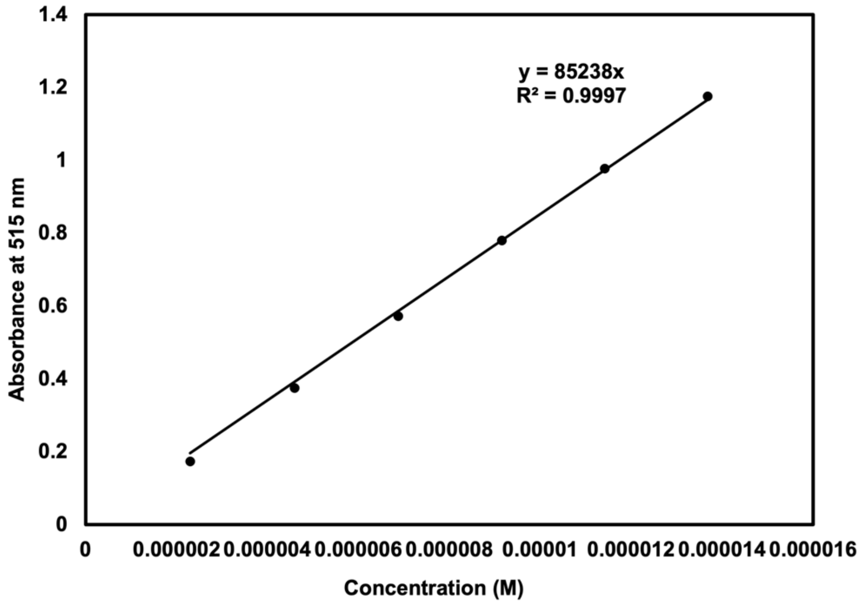

Figure S19. Calibration curve for the determination of the molar extinction coefficient for PDIN-FB.

Extinction coefficient: $A = \varepsilon cl$

$y = \varepsilon xl$, $l = 1$ cm

$\varepsilon = 85{,}238\ M^{-1}\,cm^{-1}$

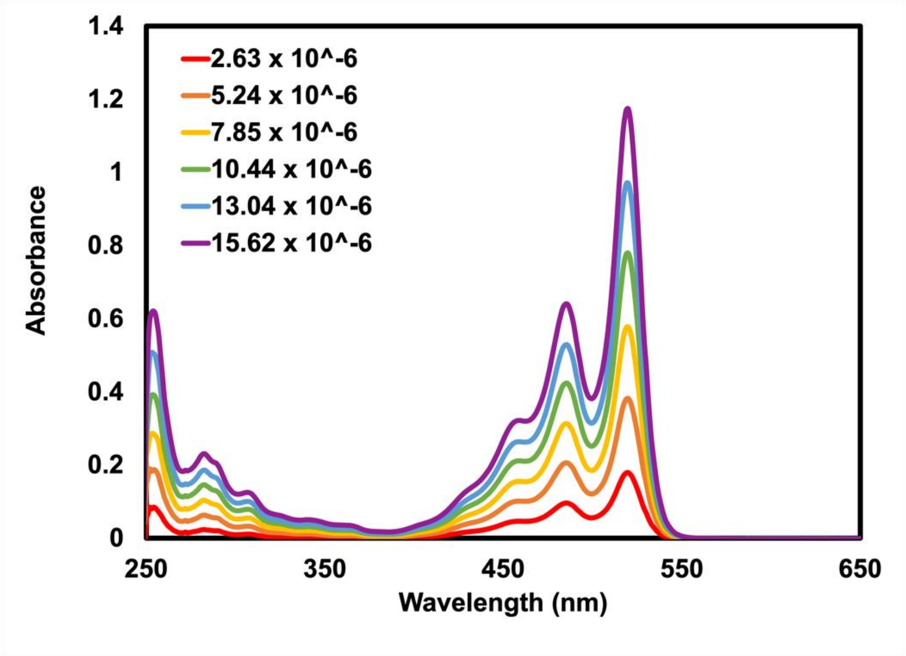

Figure S20. UV–visible spectra of PDIN-B in ethyl acetate at different concentrations.

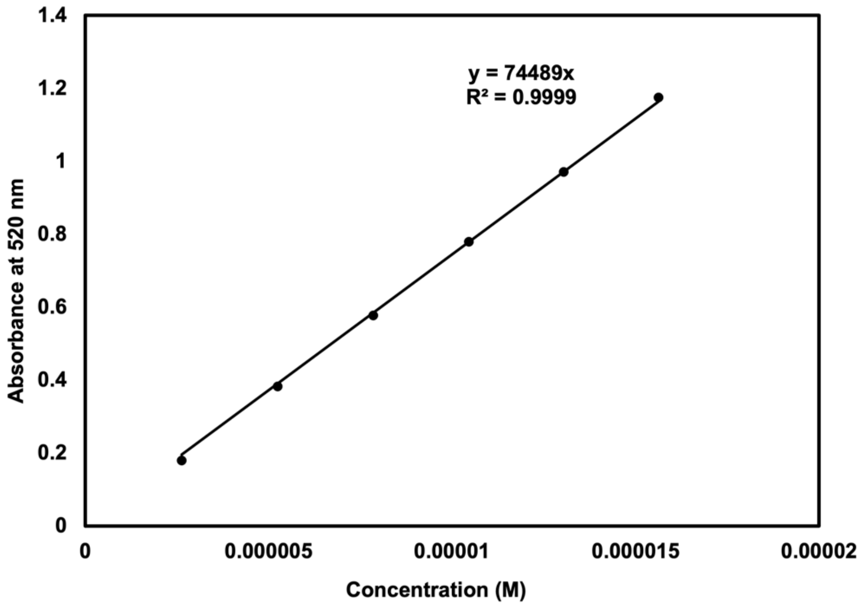

Figure S21. Calibration curve for the determination of the molar extinction coefficient for PDIN-B.

Extinction coefficient: $A = \varepsilon cl$

$y = \varepsilon xl$, $l = 1$ cm

$\varepsilon = 74{,}489\ M^{-1}\,cm^{-1}$

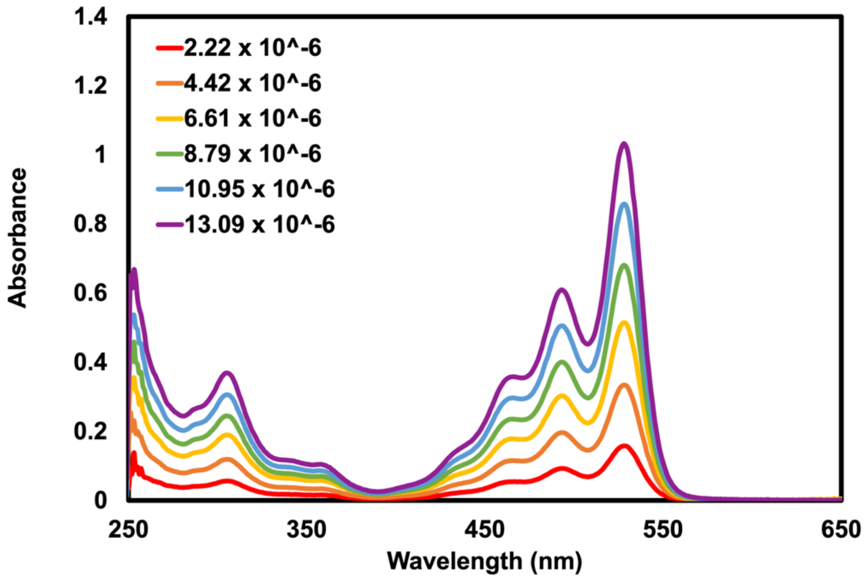

Figure S22. UV–visible spectra of CN-PDIN-FB in ethyl acetate at different concentrations.

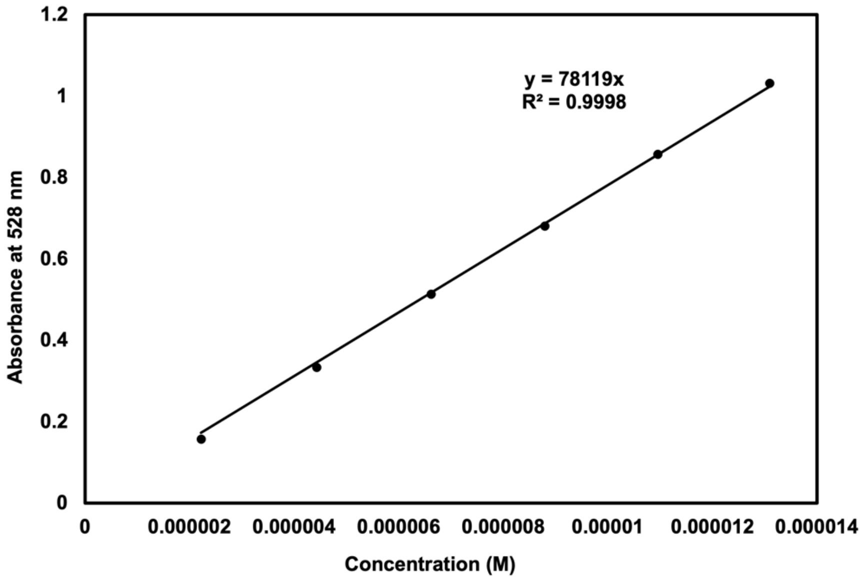

Figure S23. Calibration curve for the determination of the molar extinction coefficient for CN-PDIN-FB.

Extinction coefficient: $A = \varepsilon cl$

$y = \varepsilon xl$, $l = 1$ cm

$\varepsilon = 78{,}119\ M^{-1}\, cm^{-1}$

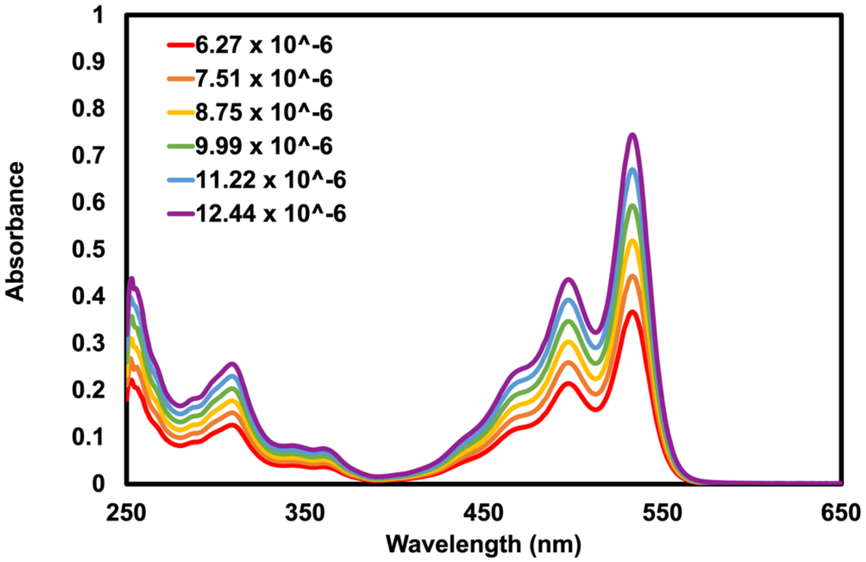

Figure S24. UV–visible spectra of CN-PDIN-B in ethyl acetate at different concentrations.

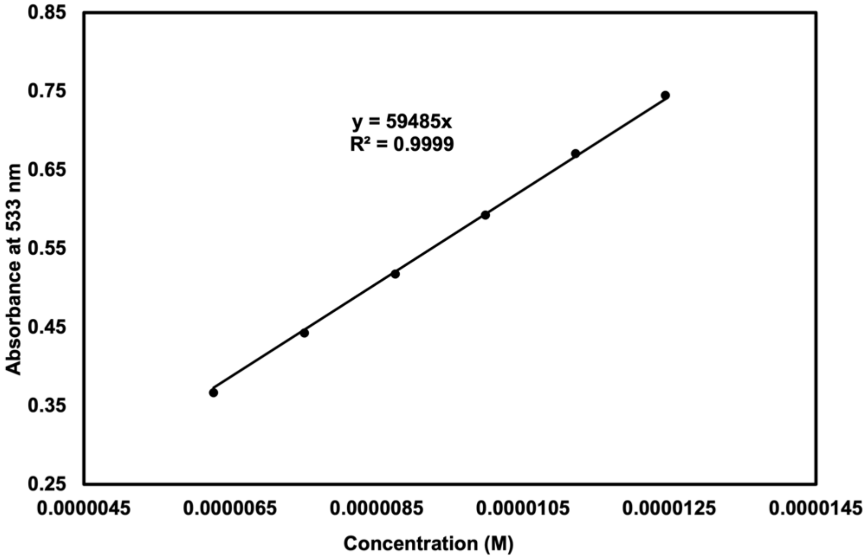

Figure S25. Calibration curve for the determination of the molar extinction coefficient for CN-PDIN-B.

Extinction coefficient: $A = \varepsilon cl$

$y = \varepsilon xl$, $l = 1$ cm

$\varepsilon = 59{,}485\ M^{-1} cm^{-1}$

8. Solution Processing

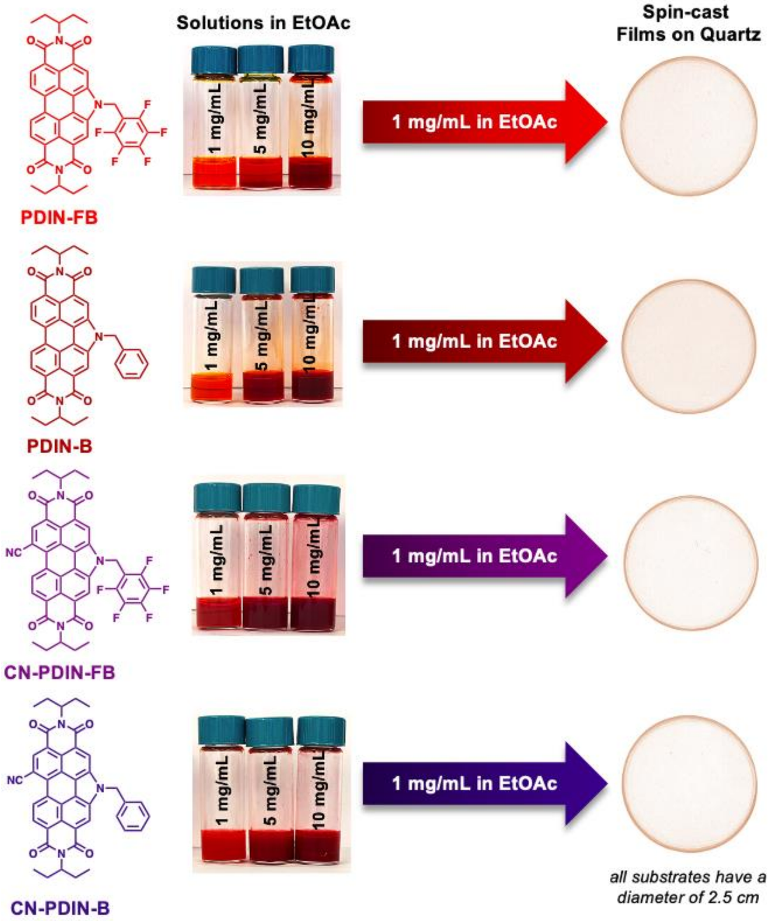

Figure S26. PDIN-FB, PDIN-B, CN-PDIN-FB, and CN-PDIN-B (structures on the left) can be solubilized in ethyl acetate at concentrations of 1 mg/mL (pictures of vials in the middle). At higher concentrations the solution become opaque. Solutions at 1 mg/mL can be cast onto quartz substrates giving uniform thin films as pictured on the right. The photographs depicted in Figure S26 were created by Kathryn Wolfe, have been used for the first time in this publication, and have not been published previously with copyright transfer to another publisher.

9. Differential Pulse Voltammetry

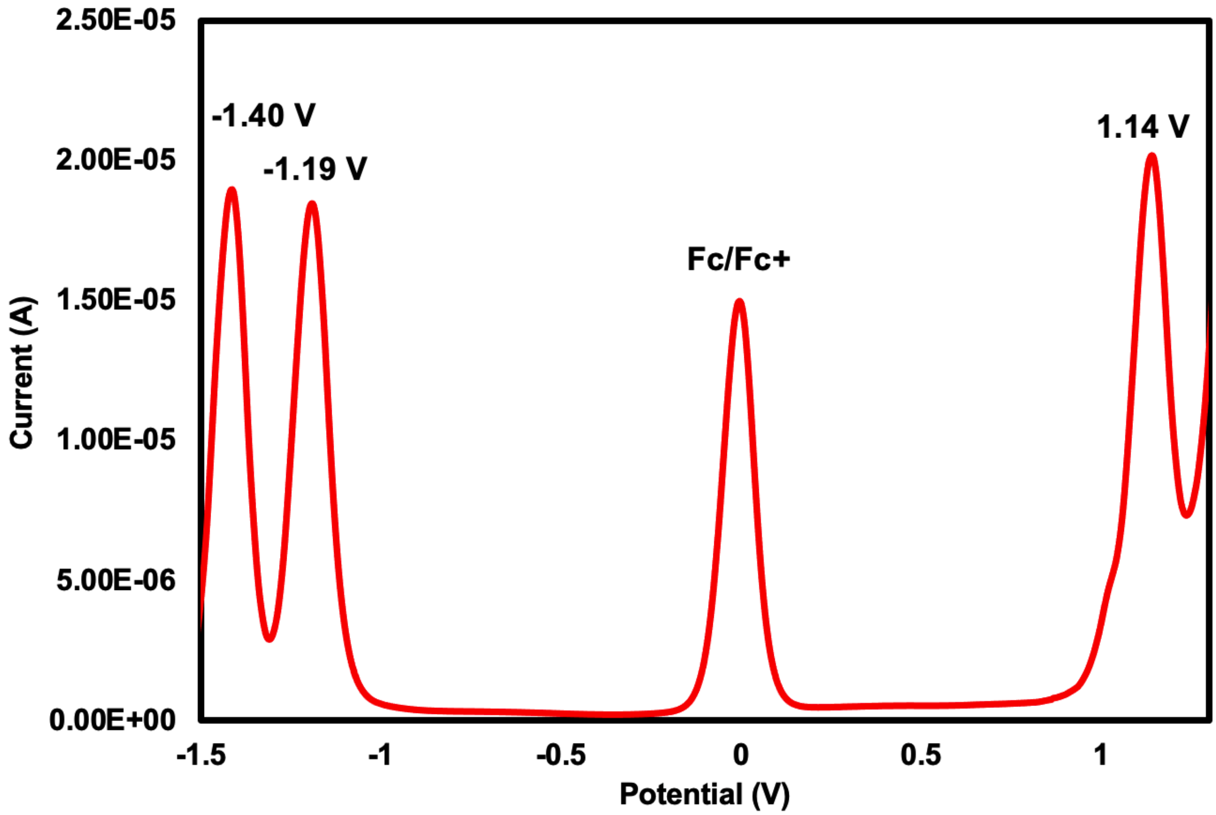

Figure S27. Differential Pulse Voltammogram for PDIN-FB.

Data corresponds to a HOMO of −5.9 eV and a LUMO of −3.6 eV (in agreeance with CV data).

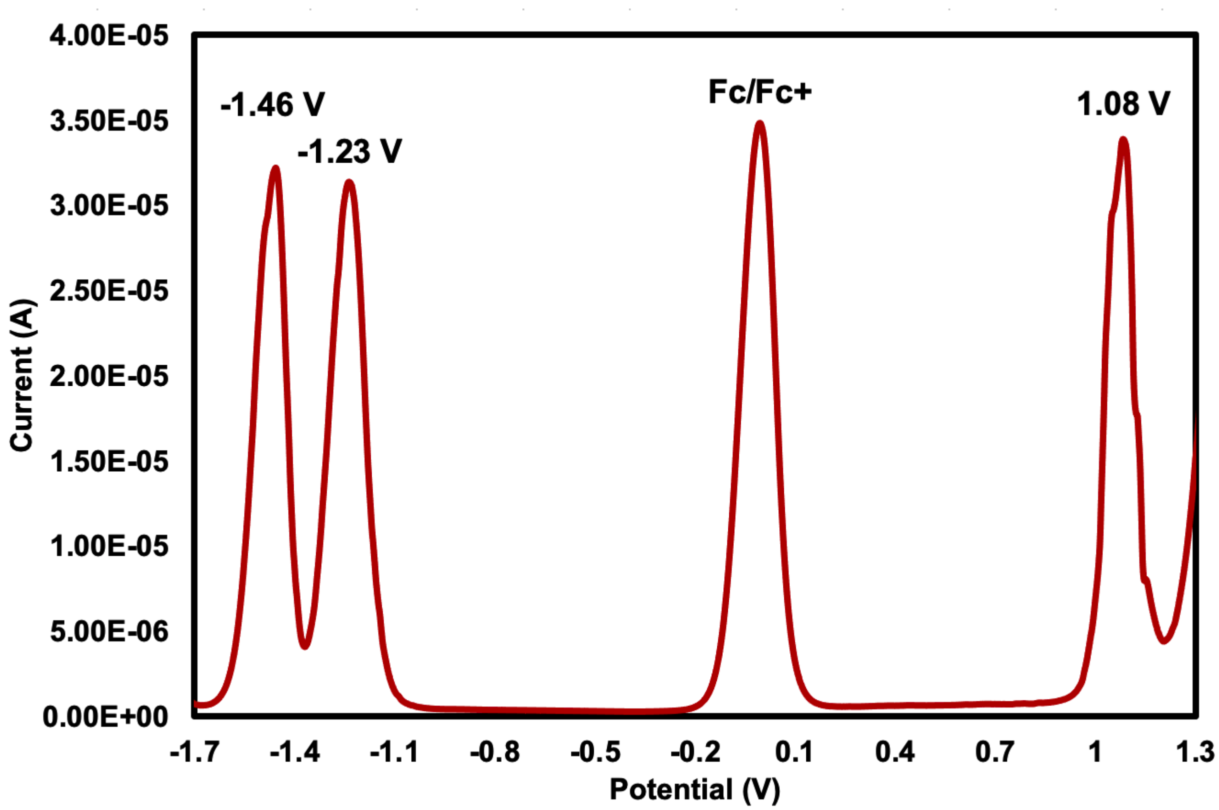

Figure S28. Differential Pulse Voltammogram for PDIN-B.

Data corresponds to a HOMO of −5.9 eV and a LUMO of −3.6 eV (in agreeance with CV data).

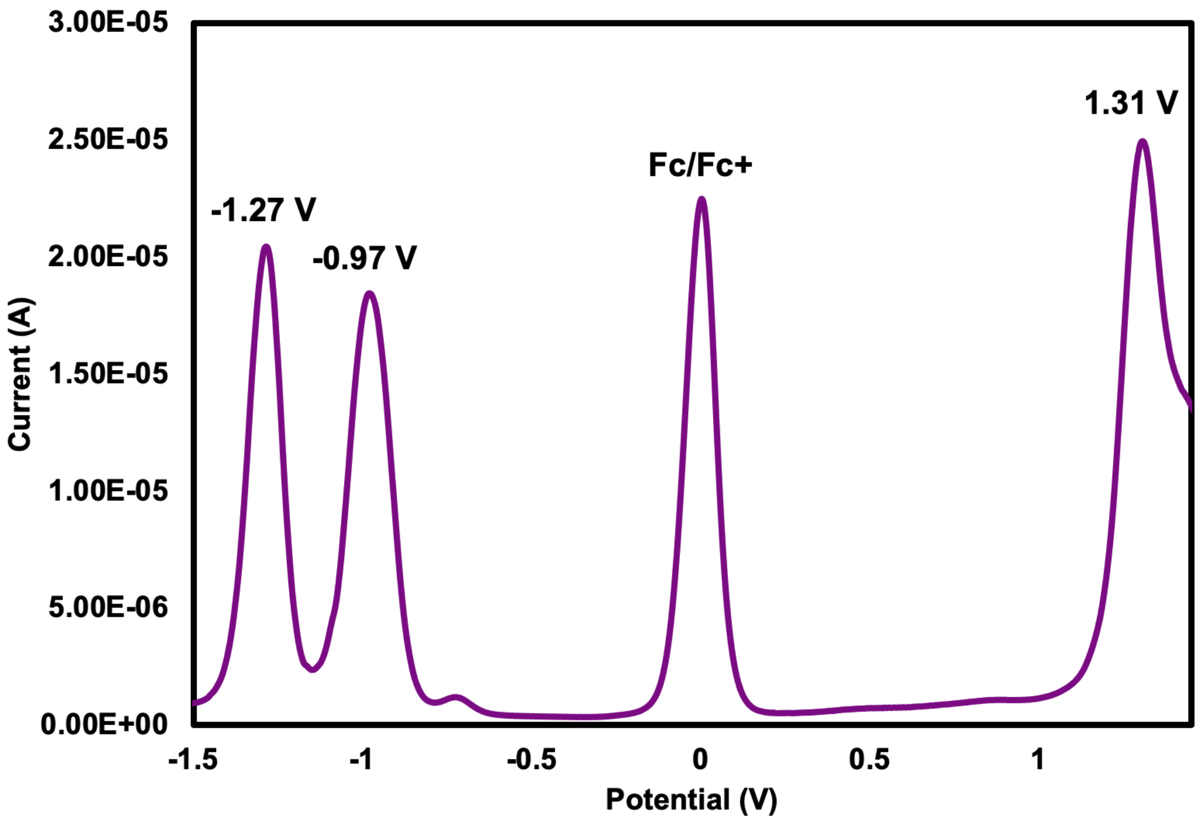

Figure S29. Differential Pulse Voltammogram for CN-PDIN-FB.

Data corresponds to a HOMO of −6.1 eV and a LUMO of −3.8 eV (in agreeance with CV data).

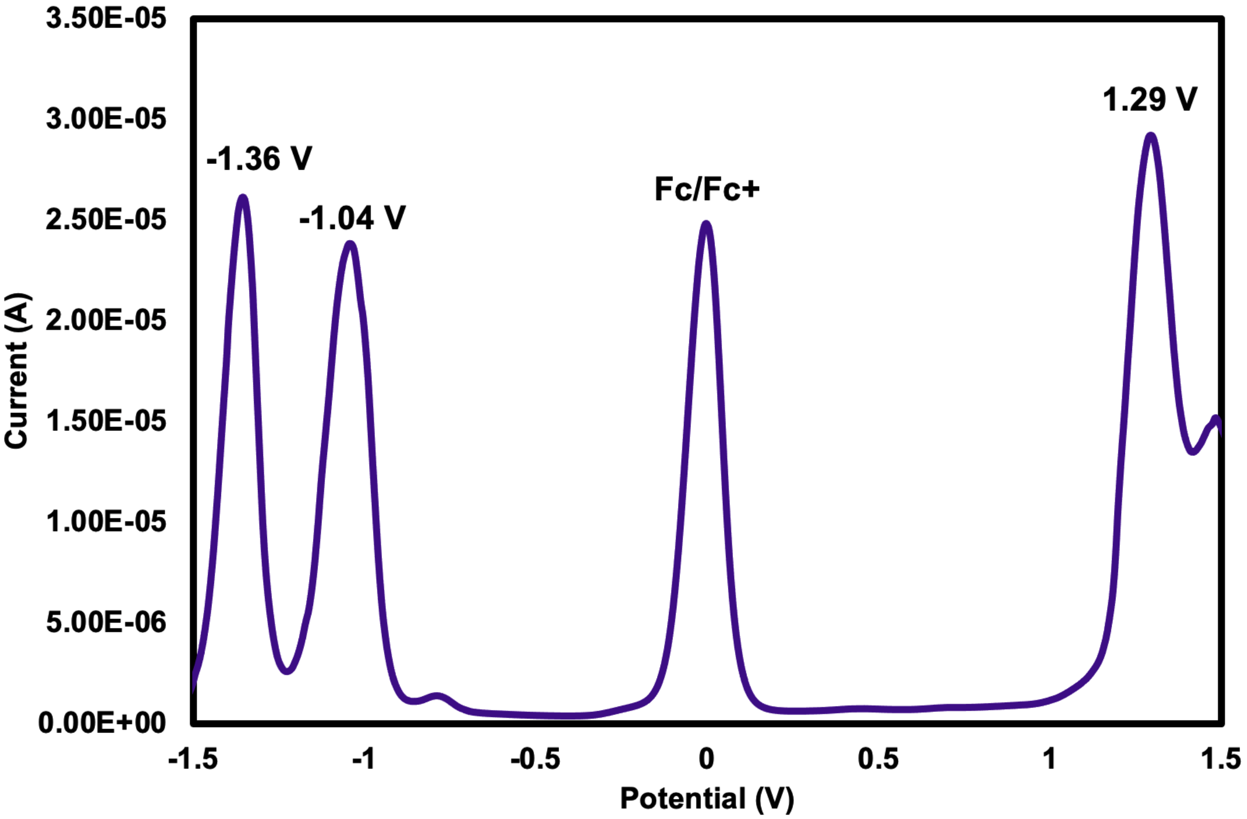

Figure S30. Differential Pulse Voltammogram for CN-PDIN-B.

Data corresponds to a HOMO of −6.1 eV and a LUMO of −3.8 eV (in agreeance with CV data).

10. Integrated J_{SC} from EQE

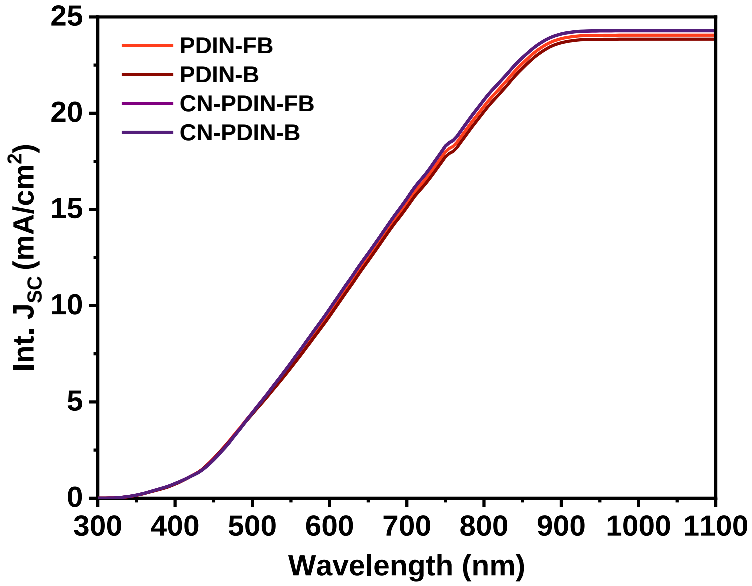

Figure S31. Integrated J_{SC} calculated from external quantum efficiency measurements of the solar cells.

11. Statistical Evaluation of PV Parameters

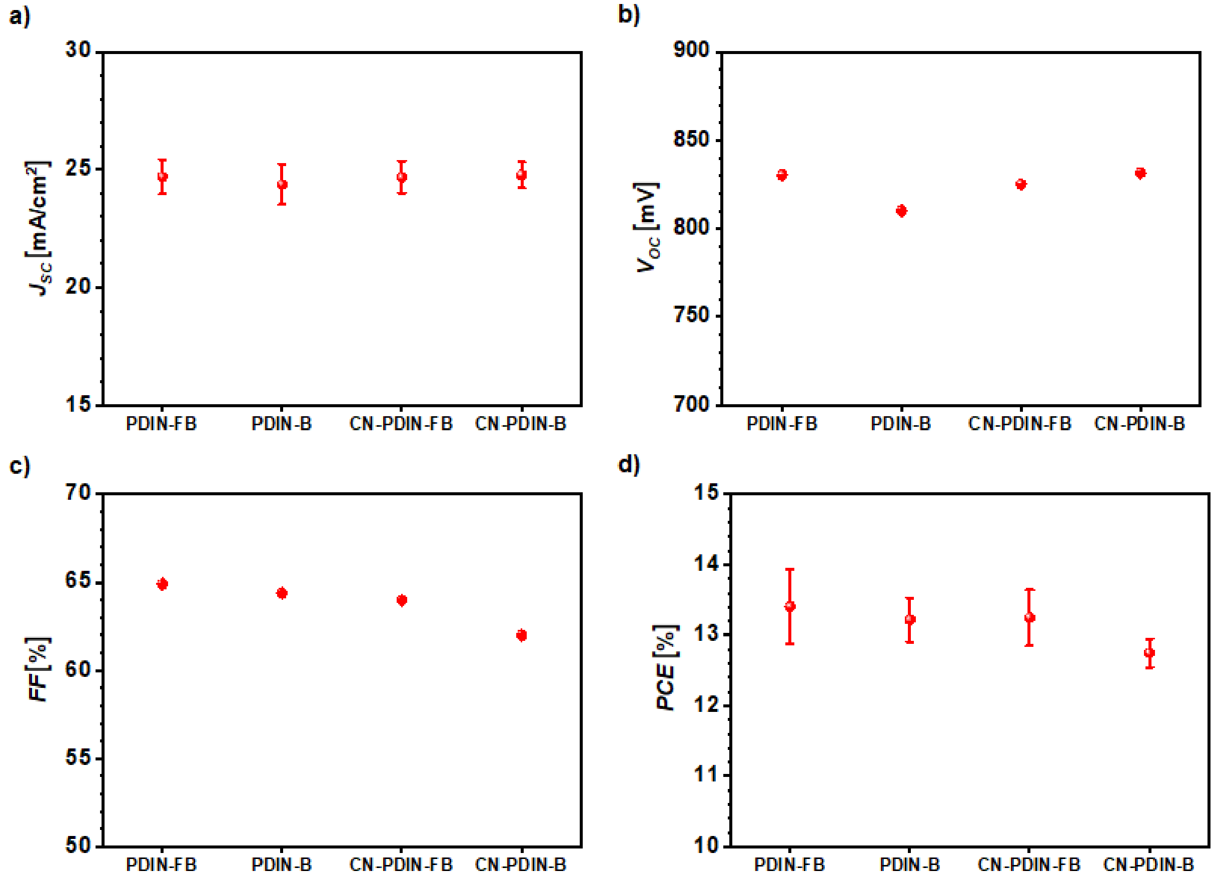

Figure S32. Statistical evaluations of photovoltaic parameters (J_{sc}, V_{oc}, FF, PCE) of PM6:Y6 devices in conventional architecture.

Table S1. Average photovoltaic parameters and standard deviation. Here short circuit current is J_{SC}; open circuit voltage is V_{OC}; power conversion efficiency is η. The averages were taken from 10 different devices.

CIL	J_{SC} (mA/cm²)	V_{OC} (mV)	FF (%)	η (%)
**PDIN-FB**	24.70 ± 0.73	830 ± 0.004	64.9 ± 0.02	13.41 ± 0.53
**PDIN-B**	24.38 ± 0.86	810 ± 0.091	64.4 ± 0.01	13.22 ± 0.32
**CN-PDIN-FB**	24.69 ± 0.67	825 ± 0.004	64.0 ± 0.01	13.25 ± 0.39
**CN-PDIN-B**	24.78 ± 0.54	831 ± 0.003	62 ± 0.01	12. 75 ± 0.21

12. Light *J–V* characteristics

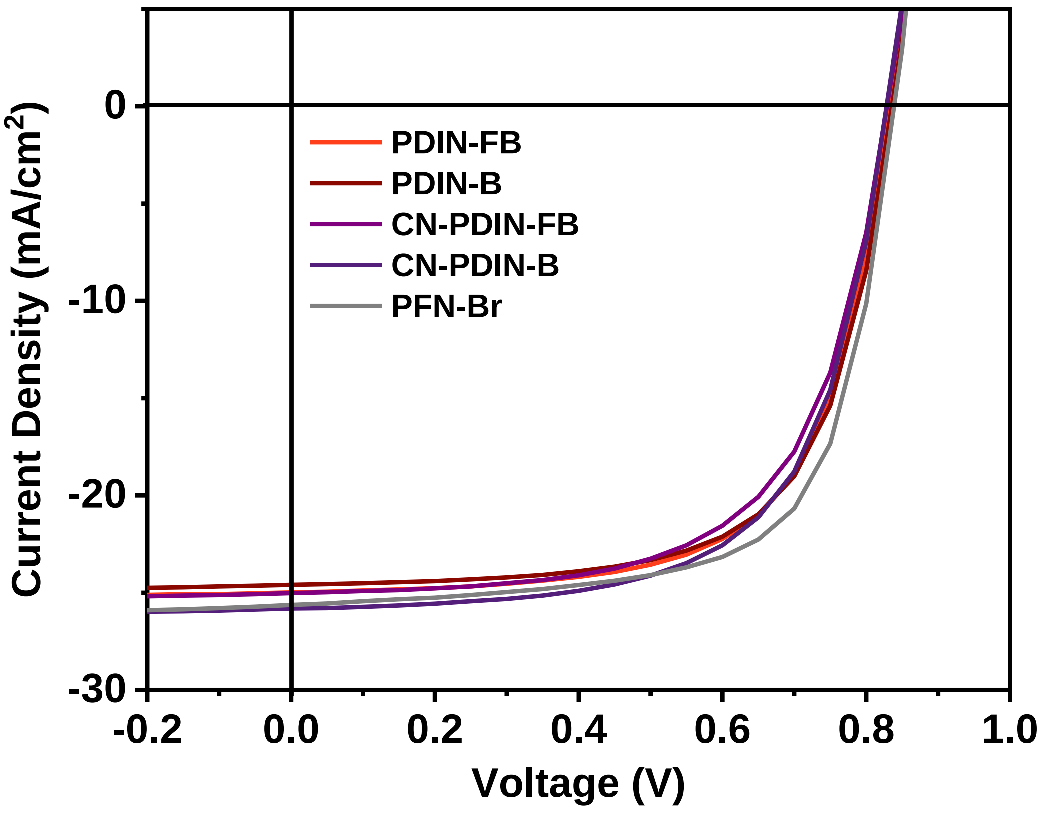

Figure S33. Current density-voltage characterization under illumination using N-annulated perylene diimides-based CILs compared with the commonly used PFN-Br (grey line) for PM6:Y6 solar cells.

13. Calculation of Extraction Efficiency

The extraction probability was calculated from the dark and light IV curves following the procedure.

First, the photocurrent density was calculated:

$$J_{\mathrm{ph}} = J_{\mathrm{L}} - J_{\mathrm{D}},$$

with J_{L} being the light current density and J_{D} being the dark current density.

Then we determined the built-in potential. V_{bi}, which is equal to the voltage at zero current photo density $\left(V_{\mathrm{bi}} = V\left(J_{\mathrm{ph}} = 0\right)\right)$.

From this, we then calculated the potential across the solar cell:

$$V = V_{\mathrm{bi}} - V_{\mathrm{appl}},$$

with V_{appl} being the externally applied voltage over the solar cell.

Finally, to calculate the extraction efficiencies at short circuit current density $\left(P_{J_{\mathrm{SC}}}\right)$ and maximum power point (P_{MPP}), we calculated the ratio between the $J_{\mathrm{ph}}\left(V_{\mathrm{appl}} = 0\right)$ to J_{ph} at saturation or respectively $J_{\mathrm{ph}}\left(V_{\mathrm{appl}} = V_{\mathrm{MPP}}\right)$.

14. Light Intensity Dependent *J–V*

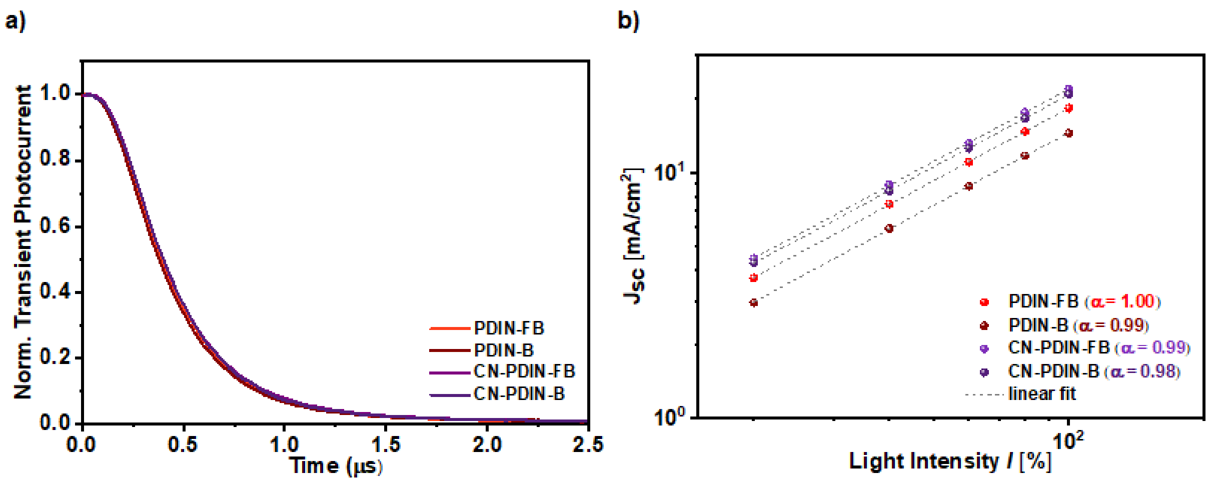

Figure S34. a) Normalized transient photocurrent measurement, b) short circuit current density vs light intensity measurements. The values of alpha are shown with the legend inside the graph, where slightly lower J_{SC} compared to the J_{SC} obtained by IV and EQE measurements are due to the different intensities of the PAIOS tools.

15. References

(1) Cardona, C. M.; Li, W.; Kaifer, A. E.; Stockdale, D.; Bazan, G. C. *Adv. Mater.* **2011**, *23*, 2367–2371. https://doi.org/10.1002/adma.201004554.
